# Dysregulation of epicardial adipose tissue in cachexia due to heart failure: the role of natriuretic peptides and cardiolipin

**DOI:** 10.1002/jcsm.12631

**Published:** 2020-10-20

**Authors:** Petra Janovska, Vojtech Melenovsky, Michaela Svobodova, Tereza Havlenova, Helena Kratochvilova, Martin Haluzik, Eva Hoskova, Terezie Pelikanova, Josef Kautzner, Luca Monzo, Ivana Jurcova, Katerina Adamcova, Lucie Lenkova, Jana Buresova, Martin Rossmeisl, Ondrej Kuda, Tomas Cajka, Jan Kopecky

**Affiliations:** ^1^ Department of Cardiology Institute for Clinical and Experimental Medicine ‐ IKEM Prague Czech Republic; ^2^ Institute of Physiology of the Czech Academy of Sciences Prague 4 Czech Republic

**Keywords:** Heart failure, Lipolysis, Natriuretic peptides, Adipose tissue, Cardiolipin, Cardiac cachexia

## Abstract

**Background:**

Cachexia worsens long‐term prognosis of patients with heart failure (HF). Effective treatment of cachexia is missing. We seek to characterize mechanisms of cachexia in adipose tissue, which could serve as novel targets for the treatment.

**Methods:**

The study was conducted in advanced HF patients (*n* = 52; 83% male patients) undergoing heart transplantation. Patients with ≥7.5% non‐intentional body weight (BW) loss during the last 6 months were rated cachectic. Clinical characteristics and circulating markers were compared between cachectic (*n* = 17) and the remaining, BW‐stable patients. In epicardial adipose tissue (EAT), expression of selected genes was evaluated, and a combined metabolomic/lipidomic analysis was performed to assess (i) the role of adipose tissue metabolism in the development of cachexia and (ii) potential impact of cachexia‐associated changes on EAT‐myocardium environment.

**Results:**

Cachectic vs. BW‐stable patients had higher plasma levels of natriuretic peptide B (BNP; 2007 ± 1229 vs. 1411 ± 1272 pg/mL; *P* = 0.010) and lower EAT thickness (2.1 ± 0.8 vs. 2.9 ± 1.4 mm; *P* = 0.010), and they were treated with ~2.5‐fold lower dose of both β‐blockers and angiotensin‐converting enzyme inhibitors or angiotensin receptor blockers (ACE/ARB‐inhibitors). The overall pattern of EAT gene expression suggested simultaneous activation of lipolysis and lipogenesis in cachexia. Lower ratio between expression levels of natriuretic peptide receptors C and A was observed in cachectic vs. BW‐stable patients (0.47 vs. 1.30), supporting activation of EAT lipolysis by natriuretic peptides. Fundamental differences in metabolome/lipidome between BW‐stable and cachectic patients were found. Mitochondrial phospholipid cardiolipin (CL), specifically the least abundant CL 70:6 species (containing C16:1, C18:1, and C18:2 acyls), was the most discriminating analyte (partial least squares discriminant analysis; variable importance in projection score = 4). Its EAT levels were higher in cachectic as compared with BW‐stable patients and correlated with the degree of BW loss during the last 6 months (*r* = −0.94; *P* = 0.036).

**Conclusions:**

Our results suggest that (i) BNP signalling contributes to changes in EAT metabolism in cardiac cachexia and (ii) maintenance of stable BW and ‘healthy’ EAT‐myocardium microenvironment depends on the ability to tolerate higher doses of both ACE/ARB inhibitors and β‐adrenergic blockers. In line with preclinical studies, we show for the first time in humans the association of cachexia with increased adipose tissue levels of CL. Specifically, CL 70:6 could precipitate wasting of adipose tissue, and thus, it could represent a therapeutic target to ameliorate cachexia.

## Introduction

Despite the fact that obesity is a strong risk factor for heart failure (HF) in the general population, obese patients with already established HF have better long‐term prognosis than their non‐obese counterparts, a situation sometimes referred to as ‘an obesity paradox’.^1^, ^2^, ^3^ In patients with advanced HF, fat mass seems to be associated with better survival^2^, ^4^ while non‐intentional weight loss, the defining feature of cardiac cachexia,^3^, ^5^, ^6^, ^7^ is a strong independent risk factor for mortality from HF.^1^, ^3^ Other chronic conditions such as cancer or chronic obstructive pulmonary disease often also lead to cachexia, which compromises treatment options and survival.^8^ Thus, distinct diseases may trigger a final common mechanism that leads to chronic body wasting and promotes other organ dysfunctions. Importantly, cachexia could not be reversed using conventional nutritional support, and effective mechanism‐based treatment of cachexia needs to be developed.^8^

Disturbed energy handling in adipose tissue represents an early event in the development of cancer cachexia,^8^, ^9^, ^10^ but the role of metabolic, immune, and secretory alterations of adipose tissue in the development and/or progression of cardiovascular diseases (including HF) is poorly understood.^11^, ^12^, ^13^ In particular, epicardial adipose tissue (EAT) was in the focus of very few studies.^12^, ^13^, ^14^ Due to its close proximity and shared microvascular network, the EAT‐myocardium microenvironment can be a place of intensive exchange of paracrine regulators or metabolic substrates, and it may serve as a transducer of systemic metabolic state, inflammation, or neurohumoral activation on the cardiac muscle.^15^, ^16^

Fatty acids released from adipose tissue are crucial for cardiac function, because a majority of cardiac ATP comes from fatty acid β‐oxidation and oxidative phosphorylation in mitochondria.^17^ Activation of fatty acid metabolism is common in advanced stages of HF, due to enhanced adipose tissue lipolysis.^11^ Lipolysis is activated in HF due to activation of the sympathetic nervous system, renin‐angiotensin‐aldosterone system (RAAS)^17^, ^18^, ^19^, ^20^ and elevated circulating levels of natriuretic peptides A and B (ANP and BNP)^11^, ^18^ that are secreted directly by the heart.^18^, ^19^, ^21^ Adipose tissue inflammation and insulin resistance further contribute to altered adipose tissue metabolism in HF patients.^8^, ^17^ In advanced HF, excessive fatty acid mobilization leads to a switch in substrate utilization by the myocardium, from β‐oxidation to energetically less effective glycolysis,^17^, ^22^ and eventually to oxidation of ketone bodies.^22^

In order to identify potential therapeutic targets to reduce body wasting and improve HF outcomes, we conducted a study that compared clinical characteristics, circulating markers, and notably, EAT gene expression and metabolome in advanced HF patients with or without cardiac cachexia.

## Materials and methods

### Study cohort

Study cohort (*n* = 52) consisted of consecutive patients with end‐stage HF who underwent heart transplantation at the Institute for Clinical and Experimental Medicine (IKEM) in Prague, Czech Republic. Subjects with body weight (BW)‐reducing therapy or endocrine disease were excluded. Samples of EAT from the HF patients were harvested from the anterior interventricular groove immediately after explantation of the heart and were placed into liquid nitrogen and stored at −80°C until analysis. BW‐trajectories during the last 6 months before transplantation were obtained by direct questioning and from medical records. Cachexia was defined as non‐intentional non‐edematous BW loss of at least 7.5% during the previous 6 months, as compared with BW‐stable patients.^3^ This relatively high cut‐off (other studies^3^, ^5^, ^6^, ^7^) was used in order to maximally unmask the impact of BW‐wasting on various parameters analysed using a relatively small patient's cohort (Discussion).

Medical records were queried to obtain clinical history, echocardiographic, and haemodynamic examinations obtained before heart transplantation. The daily dose of β‐adrenergic blockers was converted to equivalents dose of metoprolol (mg/day) using ratios of guidelines‐recommended target doses (Table 7.2 in Ponikowski *et al*.^6^). In similar way, daily dose of RAAS activity inhibitors, namely angiotensin‐converting enzyme (ACE) inhibitors or angiotensin receptor blockers (ARB), was converted to equivalent dose of ramipril (mg/day). None of the patients was treated by sacubitril/valsartan. The protocol was approved by the Ethics Committee of IKEM. All the patients signed informed consent approving their enrolment in the study and research analysis of the EAT and blood samples.

### Epicardial adipose tissue gene expression

Analysis was performed using total RNA isolated from the tissue and quantitative real‐time PCR. Results were normalized to geometric mean of three housekeeping genes: GAPDH, HPRT, and PPIA. For gene names, PCR primers and other details, see Supporting Information, *Table* S1.

### Blood tests

Serum and plasma samples were stored at −80°C. Routine biochemical parameters were evaluated using an automated Abbott Architect ci1600 analyser. BNP concentrations were measured using a microparticle immunoassay (Architect BNP; Abbott Laboratories). Insulin was measured using RIA (Beckman‐Coulter).

### Metabolomic and lipidomic analysis

A combined targeted and untargeted workflow for the metabolome, lipidome and exposome characterization was performed using EAT extracts (Supporting Information, *Method* S1).

### Statistical analyses

The data are presented as means ± standard error or means ± standard deviation, as indicated. Comparisons were analysed using Student's *t*‐test, and considered significant when *P* ≤ 0.05. Spearman's rank correlation coefficients and linear regression were used to evaluate correlations between various parameters. To find fundamental relations between data sets, partial least squares discriminant analysis was performed using MetaboAnalyst 4.0 web portal.^23^

## Results

### Clinical characteristics

The baseline characteristics of the patients are summarized in *Table* 1. The cohort consisted of predominantly middle‐aged males, with prevailing non‐ischemic HF aetiology. Twelve patients (23%) were treated for type 2 diabetes, 22 (42%) were overweight, and 6 (12%) were obese [body mass index (BMI) > 30 kg.m^−2^]. Seventeen patients (33%) reported significant (**≥**7.5%) non‐intentional BW loss during the last 6 months—the defining feature of cardiac cachexia,^4^ which discriminated them from BW‐stable patients (*n* = 35; 67%). Cachectic patients were more symptomatic, had higher New York Heart Association class and ~1.4‐fold lower EAT thickness, and tended to have lower systemic blood pressure and cardiac output. Cachectic patients had more often non‐ischemic HF aetiology and were treated with ~2.5‐fold lower dose of β‐blockers and ~2.4‐lower dose of ACE/ARB‐inhibitors as compared with BW‐stable patients. Cachectic vs. BW‐stable HF‐patients displayed ~1.4‐fold higher BNP plasma levels reflecting the higher myocardial stress, despite there was no significant difference in cardiac structure by echocardiography. Levels of plasma glucose tended to be lower, and that of haemoglobin A1C were lower in the cachectic patients (*Table* 2).

**TABLE 1:** Clinical variables and medication

Variable	All patients	BW‐stable	Cachexia	*P* value
	*n* = 52	*n* = 35	*n* = 17	
Age	54 ± 11	55 ± 10	52 ± 14	0.725
Gender (M; %)	83	86	76	0.409
Body mass index (kg/m^2^)	25 ± 4	26 ± 4	22 ± 3	**<0.001**
Body weight (BW) change (%)	−4.8 ± 6.1	−1.3 ± 3.3	−11.9 ± 4.1	**<0.001**
NYHA class (1–4)	3.4 ± 0.5	3.3 ± 0.5	3.6 ± 0.5	**0.031**
Non‐ischemic HF (%)	65	57	82	0.073
HF duration (years)	5.2 ± 4.1	5.4 ± 3.9	4.7 ± 4.4	0.556
Inotrope therapy (prior Tx; %)	21	14	35	0.082
Atrial fibrillation on ECG (%)	30	28	32	0.499
**Echocardiography**				
LV ejection fraction (%)	21 ± 6	21 ± 7	19 ± 5	0.302
LV end‐diastolic diameter (mm)	74 ± 10	72 ± 9	73 ± 12	0.794
Mitral regurgitation (0–4)	2.4 ± 1.1	2.3 ± 1.1	2.6 ± 1.1	0.307
Left atrial volume index (mL/m^2^)	74 ± 30	69 ± 29	79 ± 32	0.220
Right atrial area (cm^2^)	25 ± 8	25 ± 7	26 ± 9	0.904
Tricuspid regurgitation (grade 0–4)	2.1 ± 0.9	2.0 ± 1.0	2.1 ± 0.9	0.751
RV diastolic diameter (mm)	46 ± 8	45 ± 8	47 ± 9	0.407
RV dysfunction grade (0–4)	1.6 ± 1.0	1.7 ± 1.0	1.4 ± 0.9	0.403
TAPSE (mm)	14 ± 5	14 ± 5	14 ± 4	0.736
Epicardial fat thickness (mm)	2.6 ± 1.3	2.9 ± 1.4	2.1 ± 0.8	**0.010**
**Haemodynamics**				
Heart rate (bpm)	77 ± 13	77 ± 13	77 ± 13	0.710
Mean blood pressure (mmHg)	85 ± 10	88 ± 10	79 ± 7	0.130
Cardiac output (L/min)	3.5 ± 0.8	3.7 ± 0.7	3.2 ± 1.0	0.131
Right atrial pressure (mmHg)	9.4 ± 4.4	10.1 ± 4.9	8.1 ± 3.4	**0.043**
PA mean pressure (mmHg)	33 ± 9	34 ± 10	32 ± 8	0.384
PA wedge pressure (mmHg)	24 ± 8	24 ± 8	23 ± 7	0.563
**Medication**				
Furosemide (mg/day)	168 ± 164	155 ± 125	193 ± 226	0.534
^a^β‐blockers (mg/day)	40 ± 50	50 ± 56	20 ± 25	**0.011**
^a^ACE/ARB‐inhibitors (mg/day)	1.4 ± 2.0	1.7 ± 2.1	0.7 ± 1.3	**0.035**

β‐blockers, dose in metoprolol equivalents, ACE/ARB‐inhibitors, dose of angiotensin‐converting‐enzyme inhibitors or angiotensin receptor blockers in ramipril equivalents (Materials and methods); LV, left ventricular, RV, right ventricle; NYHA, New York Heart Association functional class; TAPSE, tricuspid annular plane systolic excursion; Tx, transplantation; PA, pulmonary artery.

Data are shown for all the patients, or two subgroups of the patients split based on their BW trajectories during the previous 6 months (BW‐stable vs. Cachexia). Data are means ± SD. Bold, statistical significant difference.

^a^ Eight out of 52 patients received neither β‐blockers nor ACE/ARB‐inhibitors, and 34 patients received a combination of the two.

[Correction added on 12 November 2020, after first online publication: Tables 1 and 2 were previously incorrect and have been replaced in this current version.]

**TABLE 2:** Plasma parameters

Parameter	^b^Control range	All patients	BW‐stable	Cachexia	*P* value
		*n* = 52	*n* = 35	*n* = 17	
^a^BNP (pg/mL)	10–80	1606 ± 1278	1411 ± 1272	2007 ± 1229	^*^ **0.010**
Creatinine (μmol/L)	64–104	109 ± 42	106 ± 31	114 ± 60	0.596
Total protein (g/L)	64–79	62 ± 6	63 ± 7	61 ± 4	0.798
C reactive protein (g/L)	< 5	9 ± 12	8 ± 9	11 ± 17	0.525
Haemoglobin (g/L)	135–175	126 ± 15	127 ± 15	124 ± 13	0.437
Total cholesterol (mmol/L)	2.9–5.1	3.7 ± 1.1	3.7 ± 1.1	3.8 ± 1.1	0.782
Triglycerides (mmol/L)	0.5–1.7	1.3 ± 0.7	1.3 ± 0.8	1.2 ± 0.5	0.415
TSH (mUI/L)	0.4–4.9	3.9 ± 3.4	3.8 ± 3.8	4.2 ± 2.5	0.803
fT3 (pmol/L)	2.9–4.9	3.4 ± 0.8	3.4 ± 0.8	3.3 ± 0.9	0.655
fT4 (pmol/L)	9–19	14 ± 4	14 ± 3	16 ± 5	0.223
Cortizol (nmol/L)	166–507	436 ± 241	451 ± 265	407 ± 187	0.493
Fasting plasma glucose (mmol/L)	3.6–5.6	5.8 ± 1.2	6 ± 1.3	5.4 ± 1.1	0.112
Fasting plasma insulin (μIU/mL)		4.1 ± 3.8	4.2 ± 4.1	3.9 ± 3.1	0.846
HOMA‐IR		1.0 ± 1.0	1.1 ± 1.1	0.9 ± 0.7	0.492
Haemoglobin A1C (mmol/mol)	20–42	49 ± 10	50 ± 12	44 ± 6	**0.017**
Bilirubin (μmol/L)	3–20	21 ± 11	21 ± 12	23 ± 11	0.584
Sodium (mmol/L)	137–144	135 ± 4	136 ± 4	134 ± 5	0.321
Potassium (mmol/L)	3.5–5.1	4.1 ± 0.6	4.1 ± 0.7	4.2 ± 0.4	0.219

Data are shown for all the patients, or two subgroups of the patients split based on BW trajectories during the previous 6 months (BW‐stable vs. Cachexia). Data are means ± SD. Bold, statistical significant difference.

^a^ Median (Q1–Q3) for BNP: all patients, 1283 (599–2413); BW‐stable, 707 (445–1815); Cachexia 1650 (902–2506).

^b^ Control (normal) range at the IKEM Central Laboratory; data are for both genders, only the BNP data are for man at 55–65 years of age, for women of this age: 10–155 pg BNP/ml plasma. HOMA, homeostasis model assessment;

^*^ For log‐transformed data.

### The impact of cachexia on epicardial adipose tissue expression of lipid metabolism, adipokines, and inflammatory genes

**FIGURE 1:**
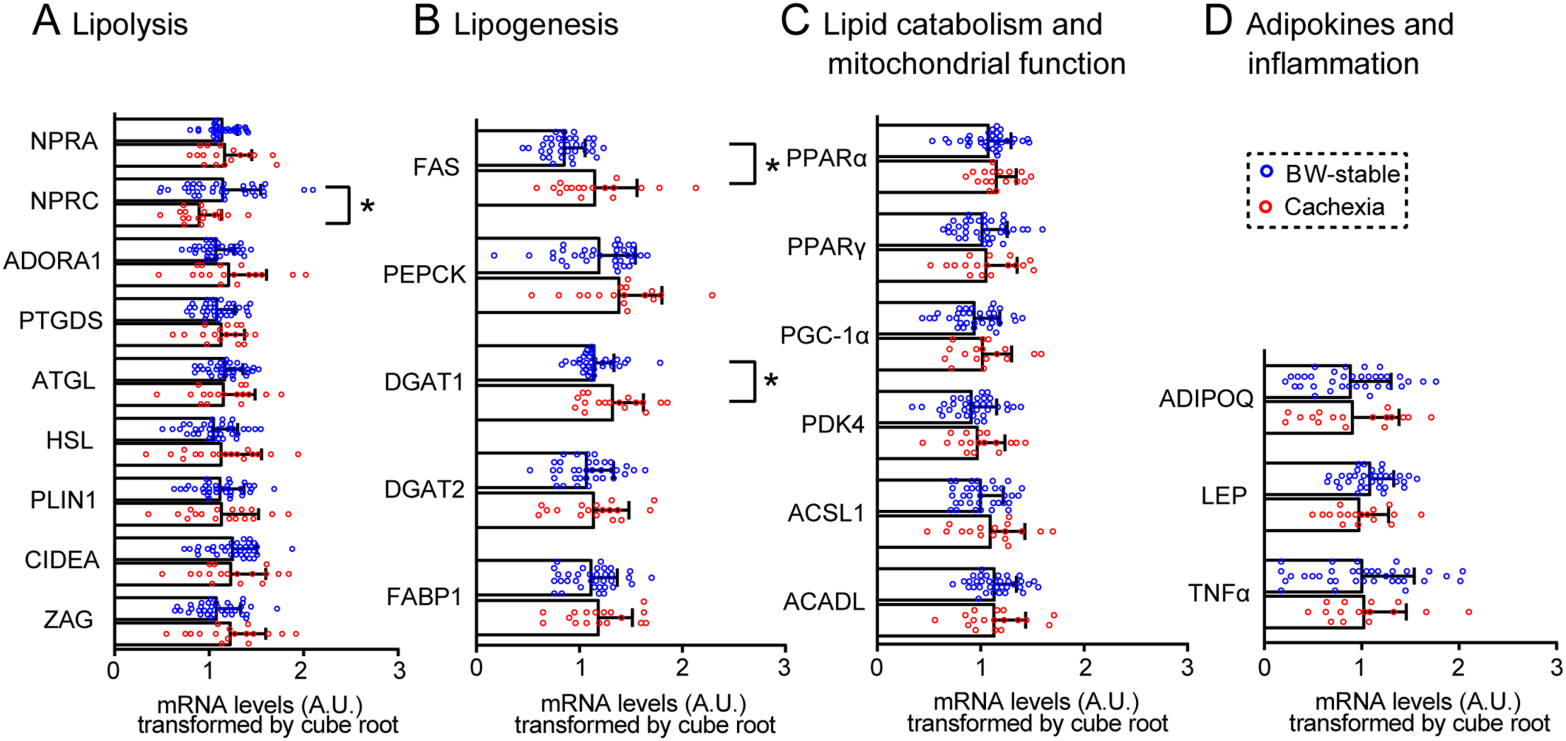
Expression of lipid metabolism, adipokines and inflammatory genes in epicardial adipose tissue. Comparison between body weight‐stable (*n* = 35) and cachectic (*n* = 17) patients. (A–D) Transcripts grouped according to their functions. Transcript levels (A.U.) were normalized (i) to geometric mean of three housekeeping genes and (ii) by cube root transformation. Data are means ± SE; Student's *t*‐test. ^*^Significant difference between the groups; for fold‐change of the means, see *Table* S2. For gene names abbreviations, see the main text and *Table* S1.

**FIGURE 2:**
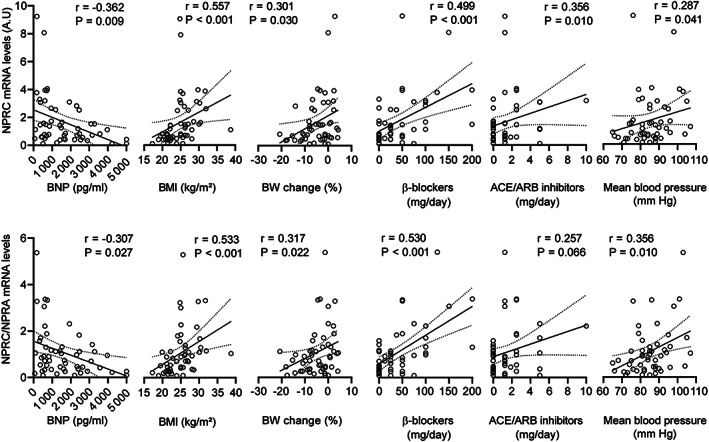
Correlation of gene expression markers of natriuretic peptide system activity with clinical parameters. Linear regression plot of the relationship between transcript levels of NPRC (upper panels), or the ratio between NPRC and NPRA transcript levels (NPRC/NPRA; lower panels), and the indicated parameters. Linear regression lines, including 95% confidence intervals, are shown. Spearman's rank correlation coefficients (*r*) and *P* value (indicated in the figures) were also calculated to assess the correlations between various parameters. For the source data, see *Figure* 1 and *Tables* S1, 1, and 2. ACE/ARB inhibitors, angiotensin‐converting enzyme inhibitors/angiotensin receptor blockers; BNP, natriuretic peptides B; BMI, body mass index; BW, body weight; NPRC, natriuretic peptide receptor C

To characterize the role of adipose tissue immunometabolism in cachexia development, expression of selected genes in EAT was evaluated and compared between BW‐stable and cachectic patients (*Figure* 1A–D; for fold change of the mean values, Supporting Information, *Table* S2). Concerning lipolysis (*Figure* 1A), expression of lipolysis‐promoting natriuretic peptide receptor A (NPRA) gene was similar in both groups (for gene names, see *Table* S1). Mean expression of natriuretic peptide receptor C (NPRC, also called clearance receptor) gene was ~2.4‐higher in BW‐stable patients. Lower NPRC/NPRA transcript level ratio in cachectic vs. BW‐stable patients (0.47 vs. 1.30; *Table S2*) suggested lower clearance of natriuretic peptides resulting in stimulation of lipolysis.^19^, ^21^ Accordingly, both NPRC expression and NPRC/NPRA transcript level ratio correlated negatively with systemic levels of BNP, positively with BMI and BW change, daily dose of both β‐blockers and ACE/ARB‐inhibitors, and mean blood pressure (*Figure* 2). Genes for both PTGDS and ADORA1 tended to be up‐regulated in association with cachexia (*Figure* 1A), suggesting increased production of prostaglandin D2 that exerted antilipolytic effect,^24^ in concert with ADORA1 stimulation.^25^ In spite of the up‐regulation of PTGDS and ADORA1, genes for (i) the key lipases ATGL and HSL,^8^ (ii) PLIN1, which is required for activation of lipolysis,^13^ (iii) cell death‐inducing DNA fragmentation factor (CIDEA), which counteracts inhibition of lipolysis by AMP‐activated protein kinase,^9^ and (iv) zinc‐α2‐glycoprotein, the lipolytic factor known to be induced in cancer cachexia,^26^ tended to be up‐regulated in cachectic patients (*Figure* 1A). With respect to genes engaged in triacylglycerols formation (*Figure* 1B), we focused on (i) FAS, which is essential for *de novo* lipogenesis, (ii) PEPCK, required for glyceroneogenesis, (iii) DGAT1 and DGAT2, engaged in fatty acid re‐esterification/triacylglycerol synthesis, and (iv) FABP1, fatty acid transporter. Mean expression of FAS, and DGAT1 was ~2.9‐fold and ~1.6‐fold higher in cachectic than BW‐stable patients, respectively. Also, expression of PEPCK, DGAT2, and FABP1 tended to be up‐regulated in cachexia. Next, we focused on expression of genes engaged in control of lipid catabolism and mitochondrial functions (*Figure* 1C). These genes included (i) PPARα, which acts both as a sensor for fatty acids and ligand‐activated transcription factor enhancing lipid catabolism, (ii) PPARγ, and its target PGC‐1α inducing mitochondrial biogenesis,^27^ (iii) PDK4, which limits glucose oxidation by inhibiting pyruvate dehydrogenase and thus supports β‐oxidation,^28^ and (iv) ACSL1 and ACADL, which are engaged in mitochondrial β‐oxidation of fatty acids. Expression of all these genes tended to be up‐regulated in cachexia (*Figure* 1C). Regarding the secretory functions and immune status of EAT (*Figure* 1D), we focused on the expression of genes for adiponectin (ADIPOQ) and leptin (LEP), representing two major adipokines,^29^ as well as pro‐inflammatory TNFα gene. Expression of none of these genes was significantly affected by cachexia.

Although ionotropic support tended to be higher in cachectic patients prior the surgery (35% vs. 14%, *Table* 1), it had no impact on the expression of lipid metabolism, adipokines, and inflammatory genes in EAT (not shown).

**FIGURE 3:**
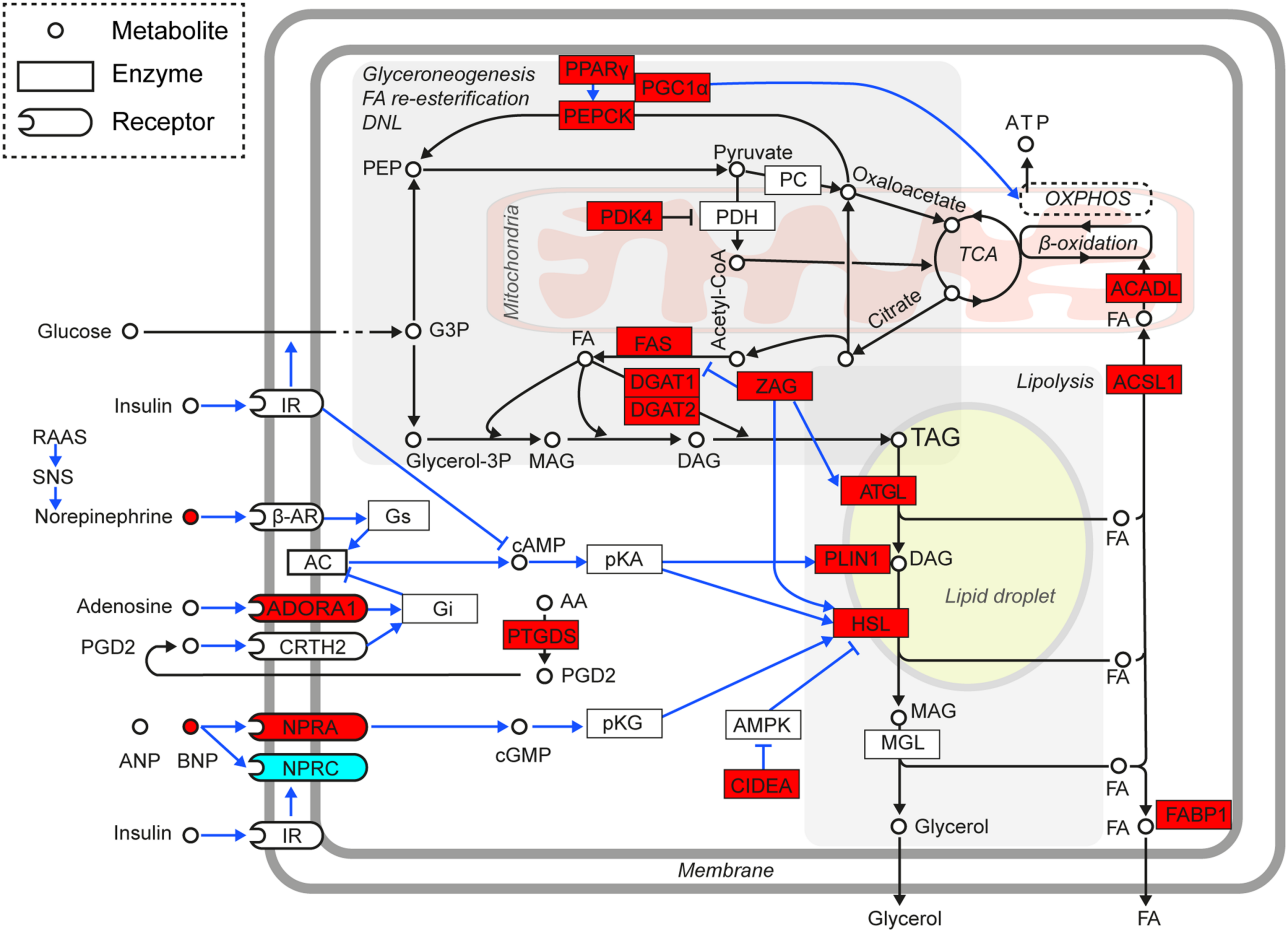
Induction of epicardial adipose tissue (EAT) lipid metabolism linked to cachexia. Gene expression data (*Figure* 1; and the FC values in *Table* S2) were collectively analysed to assess activity of the key regulatory and metabolic pathways in EAT adipocytes. Majority of the genes exerted higher mean expression levels (irrespective of the statistical significance of difference) in cachectic as compared with BW‐stable patients; also plasma levels of BNP (*Table* 2) and adenosine EAT levels (Supporting Information, *Dataset* S2) were higher in cachectic patients (red colour). Only the NPRC expression in cachectic patients was relatively low (blue colour). Data suggest that stimulation of lipolysis in EAT of HF‐patients by BNP (as well as sympathetic system and RAAS activity; Introduction; not measured here) is further augmented with adipose tissue wasting, in spite of the adaptive antilipolytic response at the PTGDS and ADORA1 gene expression level. Triacylglycerols (TAG) loss due to the increased lipolysis is not fully compensated by lipogenesis, which depends on glyceroneogenesis, *de novo* fatty acid synthesis (DNL) and fatty acid (FA) re‐esterification. Energy requirements of these anabolic pathways are covered by increased ATP production in mitochondria that combust FA (*Figure* 7) and other energy fuels (Introduction). Metabolite fluxes and regulatory effects are indicated by black and blue lines, respectively. β‐AR, β‐adrenergic receptor; AA, arachidonic acid; AC, adenylate cyclase; ACADL, long‐chain acyl‐coenzyme A dehydrogenase, also called LCAD; ACSL1, acyl‐CoA synthetase long chain family member 1; ADORA1, adenosine A1 receptor; AMPK, AMP‐activated protein kinase; ANP, A‐type natriuretic peptide; ATGL, adipose triglyceride lipase; BNP, B‐type natriuretic peptide; cAMP, cyclic adenosine monophosphate; cGMP, cyclic guanosine monophosphate; CIDEA, cell death‐inducing DNA fragmentation factor, alpha subunit‐like effector A; CoA, coenzyme A; CRTH2, prostaglandin D2 receptor 2; DAG; diacylglycerol; DGAT1and DAGT2, diacylglycerol O‐acyltransferase 1 and 2, respectively; FABP1, fatty acid binding protein 1; FAS, FA synthase; G3P, glyceraldehyde 3‐phosphate; Gi, G protein subunit alpha i1; Glycerol‐3P, glycerol 3‐phosphate; Gs, guanine nucleotide‐binding protein G(s) subunit alpha; HSL, hormone‐sensitive lipase; IR, insulin receptor; MAG, monoacylglycerol; MGL, monoacylglycerol lipase; NPRA, natriuretic peptide receptor 1; NPRC, natriuretic peptide receptor 3, also called clearance receptor; PC, pyruvate carboxylase; PDH, pyruvate dehydrogenase; PDK4, pyruvate dehydrogenase kinase 4; PEP, phosphoenolpyruvate; PEPCK, phosphoenolpyruvate carboxykinase; PGC‐1α, peroxisome proliferative‐activated receptor y coactivator 1α; PGD2, prostaglandin D2; PKA, protein kinase A; PKG, protein kinase G; PLIN1, perilipin 1; PPARγ; peroxisome proliferator activated receptor γ; PTGDS, prostaglandin D2 synthase; RAAS, renin‐angiotensin‐aldosterone system; SNS, sympathetic nervous system; TCA, tricarboxylic acid cycle; ZAG, zinc‐binding alpha‐2‐glycoprotein 1.

Relatively small differences in the gene expression pattern between BW‐stable and cachectic patients prompted us to explore the data by using a complex pathway analysis of EAT metabolism (*Figure* 3). All the differences in mean expression of the individual genes between BW‐stable and cachectic patients were considered, irrespective of their statistical significance (*Table* S2). This approach revealed a broad induction of EAT lipid metabolism in cachexia. It can be assumed that adipose tissue wasting in cachexia is caused by a net loss of tissue triacylglycerols, which are stored in adipocytes. This implies higher stimulation of lipolytic and oxidative pathways activities, which lead to decrease in the intracellular triacylglycerol pool as compared with insufficient stimulation of glyceroneogenesis, *de novo* fatty acid synthesis and fatty acid re‐esterification/triacylglycerol synthesis that are engaged in the replenishment of the pool.

### Global impact of cachexia on epicardial adipose tissue metabolome

To get further insight into the mechanisms underlying the role of adipose tissue in the development of cardiac cachexia, we performed untargeted metabolomic and lipidomic analysis of EAT. Using six different gas chromatography–mass spectrometry platforms (*Method* S1), we annotated 750 compounds including polar metabolites, complex lipids, and various exposome compounds such as drugs (Supporting Information, *Figure* S1 and *Dataset* S1). All known from these analytes, except for drugs and other non‐physiological analytes, that is the exposome compounds (*n* = 47; Supporting Information, *Table* S3), were used for further analysis.

**FIGURE 4:**
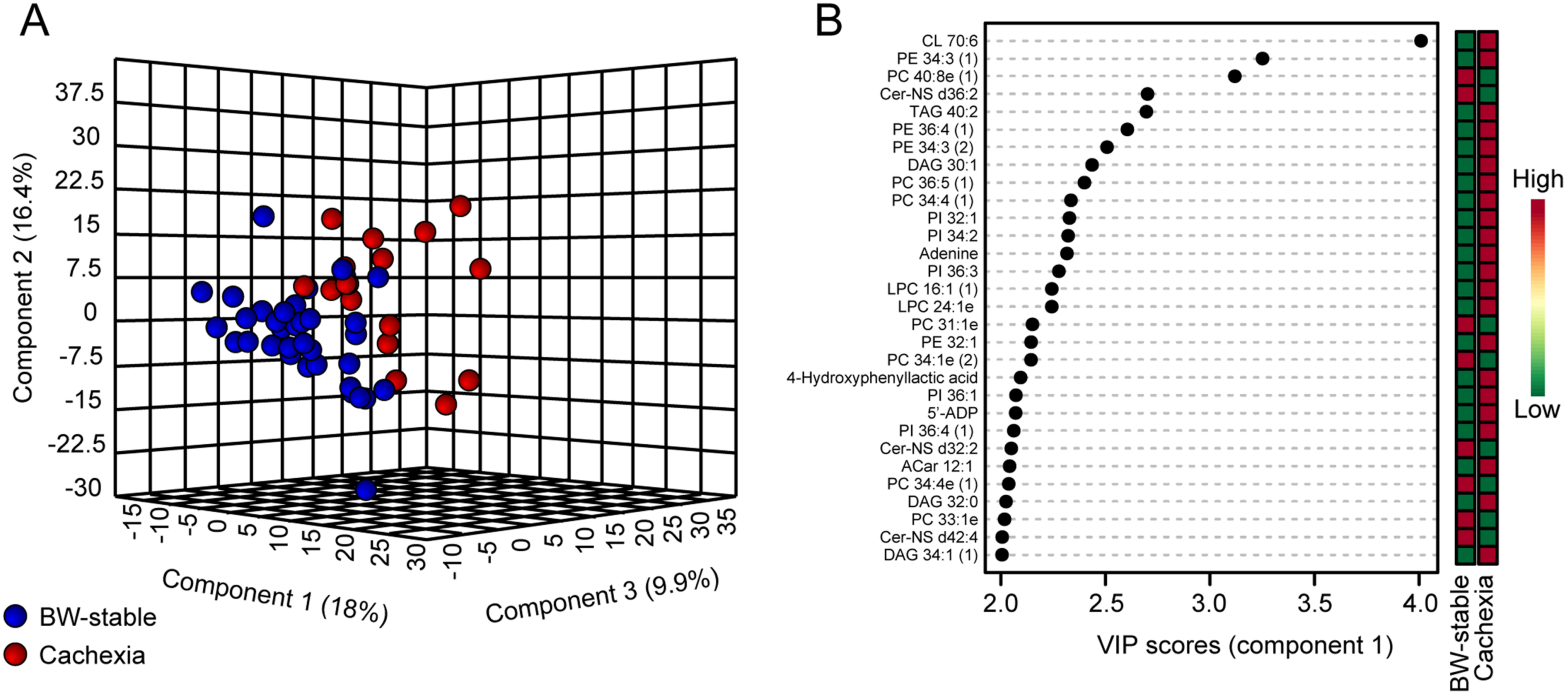
Multivariate analysis of epicardial adipose tissue (EAT) metabolome. Untargeted metabolomic and lipidomic analysis was performed using EAT extracts in all patients (*n* = 52) as described in *Method* S1. Dataset of all analytes with known structure detected in EAT (*Figure* S1), except for drugs and other non‐physiological analytes (*Table* S3), was analysed using partial least squares discriminant analysis. (A) Score plot resulting from the analysis focused on the separation between the body weight (BW)‐stable (*n* = 35) and cachectic (*n* = 17) patients (*Table* 1). (B) The corresponding variable importance in projection (VIP) plot with scores, to identify the top 30 most discriminating analytes. The coloured boxes indicate the relative concentrations of the corresponding analyte in each group; for fold‐change, see *Dataset* S2A. 5'‐ADP, adenosine‐5'‐diphosphate; Cer‐NS, ceramide non‐hydroxyfatty acid‐sphingosine; CL, cardiolipin; DAG, diacylglycerol; LPC, lysophosphatidylcholine; LPE, lysophosphatidylethanolamine; PC, phosphatidylcholine; PE, phosphatidylethanolamine; PG, phosphatidylglycerol; PI, phospatidylinositol; TAG, triacylglycerol. Lipid class x : y, acyl number of carbons : number of double bonds. Number in the brackets, isobar of the analyte.

Using partial least squares discriminant analysis, a supervised classification method, a separation between BW‐stable and cachectic patients, was observed indicating a fundamental difference between the two groups (*Figure* 4A). Variable importance in projection (VIP) identified the most discriminating analytes. Among the top 30 VIP analytes (*Figure* 4B), various (lyso)phospholipids represented a majority (60%), while triacylglycerols together with diacylglycerols were the second (13%) and ceramides the third (10%) most abundant classes among the discriminating analytes. However, cardiolipin (CL) 70:6 was clearly the single most important discriminating analyte. These results indicate that development of cachexia in HF‐patients is linked with major changes in EAT metabolome and suggest an involvement of at least some of the VIP analytes in the mechanisms underlying pathological wasting of adipose tissue. Indeed, in preclinical studies, increase of CL levels in various tissues was causally linked to cancer cachexia.^10^, ^30^, ^31^, ^32^ Therefore, CL in EAT of the HF‐patients became the major focus of the next part of our study.

### Involvement of epicardial adipose tissue cardiolipin in cachexia

**FIGURE 5:**
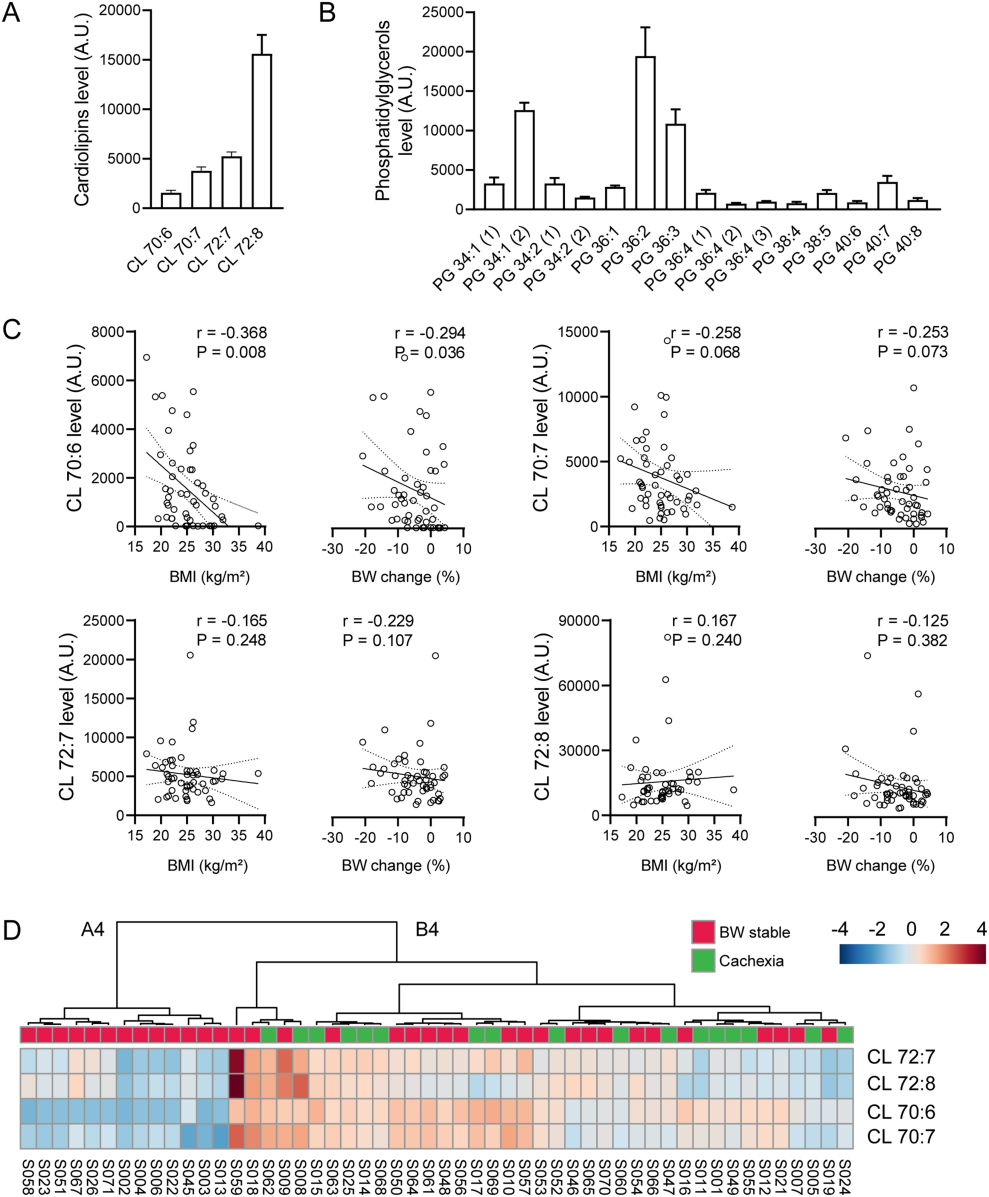
Involvement of cardiolipins (CLs) and phosphatidylglycerols (PGs) in epicardial adipose tissue (EAT) in cachexia. (A and B) Relative levels of various CLs and PGs in EAT; for the source data and further data analysis, see *Dataset* S1 and *Table* S4; data are means ± SE. (C) Linear regression plot of the relationship between (i) BMI and (ii) BW change during previous 6 months and EAT levels of various CLs. Linear regression lines, including 95% confidence intervals, are shown. Spearman's rank correlation coefficients (*r*) and *P* values (indicated in the figures) were also calculated to assess the correlations between various parameters. For the source data, see *Table* 1 and *Dataset* S1 and *Table* S4 *.* (D) Hierarchical clustering of four CLs detected in EAT. Heatmap showing data for all patients (*n* = 52). Heatmap was generated using Euclidean for distance measure and Ward for clustering algorithm. For more information about CLs, see *Table* S4. Each column represents a patient and each row represents the expression profile of an analyte across patients. BW‐stable and cachectic patients (*Table* 1) are indicated. A4 and B4, designation of two clusters separated at the first level of hierarchy.

Evaluation of the lipidomics data revealed presence of four different CLs in EAT, namely CL 70:6, CL 70:7, CL 72:7, and CL 72:8 (*Figure* 5A). Side chains of these CLs contained combinations of three different acyls, namely palmitoleic (C16:1), oleic (C18:1), and linoleic (C18:2) acid (Supporting Information, *Table* S4). Only CL 70:6 discriminated between BW‐stable and cachectic patients, while it represented the least abundant CL species (*Figure* 5A). Its levels were ~1.5‐fold higher in the cachectic patient's group (*Table* S4). EAT metabolome also contained several phosphatidylglycerol (PG) species, that is the building blocks in the synthesis of CL (*Figure* 5B). Levels of most of these PGs tended to be higher in cachectic as compared with BW‐stable patients (*Table* S4).

Importantly, EAT levels of CL 70:6 significantly correlated with BW markers, that is negatively with both BMI and BW change. Similar trends were observed with CL 70:7. No correlation between the BW markers and the levels of either CL 72:7 or CL 72:8 was observed (*Figure* 5C). Thus, the strength of the association between various CLs and the degree of cachexia increased in the case of less abundant CL species (*Figure* 5A).

Hierarchical clustering of EAT analytes using only levels of the four CLs revealed two clusters of patients at the first level of hierarchy, Cluster A4 and Cluster B4, respectively (*Figure* 5D). Cluster A4 (13 cases) was composed exclusively of BW‐stable patients. Cluster B4 (39 cases) represented a heterogeneous mixture of BW‐stable (56%) and cachectic (44%) patients. EAT levels of most CLs and PGs were higher in Cluster B4‐patients, with CL 70:6 showing the largest (~47‐fold) difference (*Table* S4). In agreement with the comparison between BW‐stable and cachectic patients (*Tables* 1 and 2), the homogenous group of BW‐stable patients in Cluster A4 exhibited lower plasma BNP levels and higher dose of both β‐blockers and ACE/ARB‐inhibitors as compared with Cluster B4‐patients (Supporting Information, *Table* S5).

At the gene expression level, Cluster A4 patients showed relatively high NPRC and relatively low ADORA1 and FAS expression, consistent with the difference in gene expression pattern between BW‐stable and cachectic patients (*Table* S2). Also minor differences in expression of the other genes between Cluster A4 and Cluster B4 patients were mostly in agreement with the corresponding differences between BW‐stable and cachectic patients; in addition, the Cluster A4 vs. Cluster B4 comparison unmasked higher expression of leptin gene in Cluster A4, that is in BW‐stable patients (*Table* S2).

The above results documented the role of CL with a specific acyl side chains composition, namely the CL 70:6 containing C16:1, C18:1, and C18:2 acyls, in the development of cardiac cachexia. Therefore, we aimed to ascertain the mechanisms underlying the differential control of EAT levels of various CLs in cachexia. First, we focused on potential role of changes in EAT levels of PGs. Multiple significant positive correlations between the levels of either CL 70:6 or CL 70:7 and most PGs were found. For CL 72:7 or CL 72:8 species, the correlations were less frequent (*Figure* S2).

**FIGURE 6:**
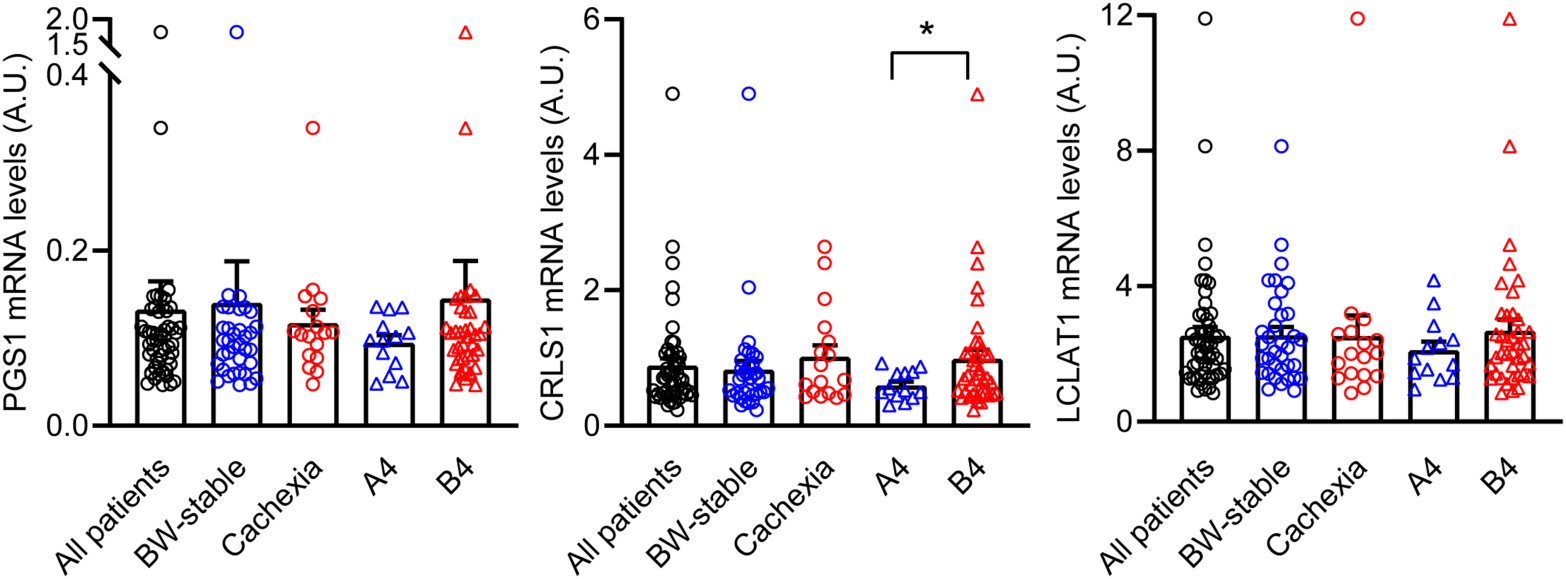
Expression of genes engaged in synthesis (PGS1 and CRLS1) and remodelling (LCLAT1) of cardiolipin across patient's subgroups. Transcript levels (A.U.) were normalized to geometric mean of three housekeeping genes. Data (*Table* S6) for all patients (*n* = 52), or the following subgroups: (i) BW‐stable (*n* = 35) and cachectic (*n* = 17) patients; and (ii) Clusters A4 and B4 patients (*n* = 13 and *n* = 39, respectively), are shown. Data are means ± SE; Student's *t*‐test. ^*^Significant difference between the groups. For gene names abbreviations, see the main text and *Table* S1.

Secondly, we focused on the role of genes engaged in CL synthesis, PG synthase 1 (PGS1) and CL synthase 1 (CRLS1), and in remodelling of the acyl side chains of CL, acyl‐CoA : lysocardiolipin acyltransferase 1 (LCLAT1). Expression of these genes was compared between BW‐stable and cachectic patients, as well as between Cluster A4 and Cluster B4 patients (*Figure* 6 and Supporting Information, *Table* S6). With PGS1 and LCLAT1, no significant differences between the groups were observed. However, CRLS1 expression was significantly higher (~1.7‐fold) in Cluster B4 as compared with Cluster A4 patients, while expression of both PGS1 and LCLAT1 showed a similar trend.

These results suggested that differential increase in the EAT levels of various CLs in cachexia could reflect both (i) basal levels of PGs that are used for CL synthesis and (ii) activity of the genes engaged in CL synthesis and remodelling.

## Discussion

To our knowledge, this is the first study focused on the role of EAT‐myocardium microenvironment in the development of cardiac cachexia. A complex approach was used, which was based on the characterization of both metabolome/lipidome and gene expression in EAT of the end‐stage HF‐patients with and without cardiac cachexia.

For the characterization of the impact of BW‐wasting on various parameters in our study, the BW‐stable and cachectic patients were discriminated using a non‐edematous BW loss of at least 7.5% during the previous 6 months. This 7.5% cut‐off, used also to define cardiac cachexia by Anker and colleagues in 1997,^3^ was higher as compared with that of 5% used in the 2008 cardiac cachexia definition^5^ or the 6% cut‐off point in the ESC Guidelines from 2016^6^; Anker *et al*.^7^ When we have analysed the data at several cut‐off points, equal to 5%, 6%, and 7.5% (not shown), we have found that the highest cut‐off (7.5%) helped to uncover subtle links between the BW‐loss and EAT gene expression (*Figure* 1), while the differences between BW‐stable and cachectic patients (i) in the clinical variables and medication (*Table* 1) or plasma parameters (*Table* 2) and (ii) metabolome/lipidome (*Figures* 4 and 5D) were only marginally affected by various cut‐off points. Thus, due to limited number of studied subjects, we choose the higher cut‐off point of BW change to enhance the contrast between cachectic and BW‐stable patients.

Our results demonstrate fundamental differences in metabolome/lipidome between BW‐stable and cachectic patients. CL, a phospholipid of the inner mitochondrial membrane, specifically CL 70:6, was the most discriminating analyte. In preclinical models of cancer cachexia, intracellular accumulation of CL led to increased energy‐wasting due to the uncoupling of mitochondrial oxidative phosphorylation.^10^, ^30^, ^31^, ^32^ This occurred in several tissues, including white fat, which became involved early during cachexia development.^10^ We demonstrate here for the first time in humans increased tissue levels of CLs in cachexia. Based on the results of the animal study,^10^ changes observed in EAT levels of CLs probably occurred also in other fat depots and other tissues of the cachectic patients. However, CL‐induced modulation of EAT metabolism could be of special importance for heart function due to the mutual interactions within the EAT‐myocardium environment (Introduction).

Our results suggest that CL of a specific acyl profile could be causally involved in cachexia. Side chains of the four CLs contained in EAT represented combinations of only three different acyls, namely C16:1, C18:1, and C18:2, with the CL species abundance increasing with larger molecular mass and a higher degree of unsaturation and length of the acyl side chains (Table *S*4). It was not the major CL species, that is CL 72:8 (tetralinoleoyl‐CL), which is normally present in insulin‐sensitive tissues,^33^ but the least abundant CL 70:6, which discriminated between BW‐stable and cachectic patients. As suggested by the results of the animal study,^32^ CL70:6 could induce the uncoupling of oxidative phosphorylation in EAT mitochondria of cachectic patients. Its specific molecular acyl composition could underlie this effect.

Indeed, the changes in the tissue content of specific CL species and or changes in the acyl profile of CL could be the cause of specific pathologies: (i) Barth syndrome, a multisystem disorder characterized by cardiomyopathy, is associated with reduced levels of CL 72:8 (tetralinoleoyl‐CL) in mitochondria, due to the defect of CL transacylase tafazzin, (ii) decrease of CL 72:8 preceded the development of HF in spontaneously hypertensive rats and correlated with a loss of mitochondrial cytochrome oxidase activity (reviewed in Saini‐Chohan *et al*.^34^), and (iii) replacement of C18:2 by docosahexaenoic acid (C22:6) in the CL side chains leads to mitochondrial dysfunction in diabetes and other metabolic diseases.^35^ The mechanism of induction of CLs tissue levels in cachexia has yet to be characterized in detail. Our results in EAT of HF‐patients show a link to increased tissue levels of PGs, the building blocks in the synthesis of CLs, suggesting an involvement of the enzymes engaged in CL synthesis and remodelling, especially the CL synthase CRLS1.

**FIGURE 7:**
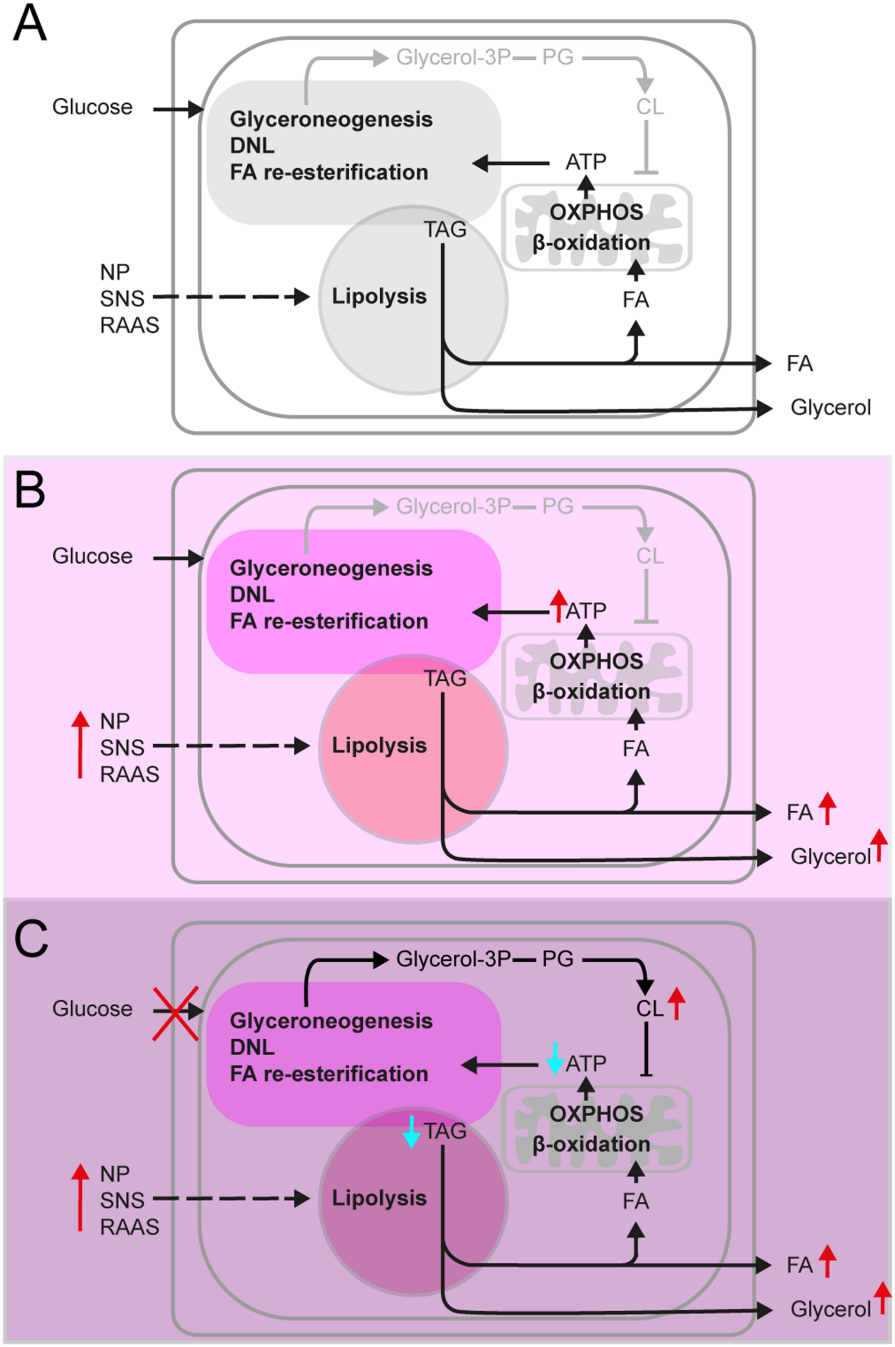
Changes in metabolism of EAT adipocytes during development of HF and cachexia. As compared with healthy subjects (A), in HF‐patients (B and C), lipid metabolism in EAT adipocytes is activated in response to elevated plasma natriuretic peptides (NP) levels and increased activity of sympathetic nervous system (SNS) and RAAS. Activation of lipolysis results in increased efflux of fatty acids (FA) and glycerol from EAT. While FA could serve as energy fuels for myocardium, glycerol is used for hepatic glyceroneogenesis. In BW‐stable HF‐patients (B), breakdown of intracellular triacylglycerols (TAG) is balanced by TAG synthesis, which depends on glyceroneogenesis, *de novo* synthesis of FA (DNL), and FA re‐esterification. These energy consuming reactions are driven by ATP, which is mostly synthesized by oxidative phosphorylation (OXPHOS) linked to β‐oxidation of FA in mitochondria. In cachectic HF‐patients (*C*), lipolysis is stimulated even more than in the BW‐stable patients, in association with elevation of intracellular CL levels. This probably results from both, increased formation of phosphatidyl glycerols and dysregulation of enzymes engaged in CL synthesis and remodelling in cachexia. Aberrantly high levels of CL, namely the CL 70:6 species, induce uncoupling of OXPHOS. Low rate of ATP synthesis limits activity of the pathways contributing to TAG synthesis, resulting in insufficient replenishment of the TAG pool in fat cells and wasting of adipose tissue. For more details, see *Figure* S3.

Previous studies focused on EAT gene expression either in patients with milder or no HF and compared subjects according to degree of left ventricular dysfunction^12^ or according to the presence of coronary artery disease.^13^ Expression of the genes characterized here remained either unaffected (LEP, TNFα; Fosshaug *et al*.^12^; and PLIN1, HSL, ATGL, ADIPOQ, LEP, IL6; Jaffer *et al*. ^13^) or only marginally changed (PPARα, IL6; Fosshaug *et al*.^12^). In this study, the overall pattern of EAT gene expression suggested simultaneous activation of lipolysis and lipogenesis in cachectic as compared with BW‐stable HF‐patients. Lipolysis was probably activated by the concert action of RAAS and sympathetic nervous systems^17^, ^18^, ^19^, ^20^ and by natriuretic peptides.^11^, ^18^ The prolipolytic factors contributing to cancer‐related cachexia, CIDEA^9^ and zinc‐α2‐glycoprotein,^26^, ^36^ were probably also involved. Activation of both lipolysis and energy dissipating fatty acid/triacylglycerol futile cycling in EAT is consistent with the high catabolic activity of adipose tissue^11^ and the overall catabolic dominance in HF‐patients^17^ that was augmented further in association with adipose tissue wasting. Similar changes in adipose tissue metabolism were found in cancer cachexia.^8^, ^9^ Presumably, the decrease in tissue triacylglycerol content due to increased lipolysis could not be compensated in full by activation of lipogenesis in EAT of cachectic patients. This is consistent with the effect of CL‐induced uncoupling of oxidative phosphorylation in adipocytes^37^, ^38^ when insufficient ATP production would limit several key biochemical activities engaged in triacylglycerols replenishment, such as glyceroneogenesis, *de novo* synthesis of fatty acids and their re‐esterification (*Figure* 7 and Supporting Information, *Figure* S3). Relatively high ADORA1 expression found in BW‐stable patients grouped in Cluster A4 is typical of EAT,^25^ and ADORA1 up‐regulation could contribute to the cardioprotection due to vasodilatation of coronary vessels^25^ and attenuation of β‐adrenergic stimulation of lipolysis.^24^ However, in general, the changes in EAT gene expression in cachexia were only mild. It should be learned if they take place also in other fat depots, which differ in their metabolic features from EAT,^12^, ^13^, ^25^ and what is the contribution of the changes in adipose tissue metabolism to cardiac cachexia. For sure, due to its small mass, EAT plays an insignificant role in total energy balance.

Our study highlights the importance of the enhanced release of natriuretic peptides from the failing heart for the changes in EAT metabolism and development of cachexia,^18^, ^19^, ^21^ as suggested by the higher levels of BNP in cachectic patients. Adipose tissue sensitivity to natriuretic peptides is regulated by NPRC and NPRC/NPRA ratio.^19^, ^21^ Hence, the relatively low NPRC/NPRA ratio in the cachectic patients, resulting in enhanced bioavailability of natriuretic peptides in the EAT‐myocardium microenvironment, could be important. In addition, a negative correlation was found between the expression of NPRC and levels of CL 70:6 in EAT (Spearman's rank correlation coefficient = −0.561), supporting the role of NPRC in CL‐induced changes of EAT metabolism in cachexia.

Comparisons between the subgroups of HF patients (i.e. BW‐stable vs. cachectic, and Cluster A4 vs. Cluster B4 patients) revealed lower plasma BNP levels and higher daily doses of both β‐blockers and ACE/ARB‐inhibitors in the patients who were resistant to cachexia. These drugs prevent adverse neurohumoral activation that accompanies HF and, thus, represent the cornerstone of effective HF therapy, with their doses usually titrated to the maximally tolerated level. Therefore, HF patients who tolerated higher doses of these drugs were better protected against the development of cardiac cachexia. Indeed, β‐adrenergic blockers and angiotensin II receptor antagonists have protective effects against fat loss due to cardiac cachexia (reviewed in Cabassi *et al*.^20^). Anticachectic, adipose tissue‐sparing effects of neurohumoral antagonist might therefore contribute to the favourable impact of these drugs on survival.

Our results support the idea that novel treatment strategies for HF‐patients may be designed to target EAT metabolism and its secretory features, while changing EAT‐myocardium microenvironment^15^ and, therefore, improving heart function. This could include neutralization of CIDEA activity to counteract excessive lipolysis in EAT, similarly as suggested for the treatment of cancer cachexia^8^, ^9^ or inhibition of ATGL activity.^14^ Our findings here open a new possibility to treat cachexia by modulating CL biosynthesis and/or CL function. To this end, the use of cell‐penetrating aromatic‐cationic tetrapeptides that selectively target CL^39^ should be explored.

In conclusion, we observed here in patients with advanced HF that BNP signalling probably contributed to changes in EAT metabolome/lipidome and gene expression, reflecting dysregulated induction of EAT lipid metabolism in cardiac cachexia. Concomitant modulation of EAT‐myocardium microenvironment may be involved in the deterioration of heart function in the cachectic patients. Our results suggest that the maintenance of stable BW and ‘healthy’ EAT‐myocardium microenvironment of the HF‐patients is associated with their ability to tolerate higher therapeutic doses of conventional therapeutics, namely neurohumoral inhibitors (ACE/ARB inhibitors and β‐adrenergic blockers). We showed that induction of EAT levels of CL 70:6 could precipitate the wasting of adipose tissue. It is likely that an induction of minor CL species underlies energy dissipation in various tissues and represents one of the key events resulting in cachexia. Our results suggest that mitochondrial CL in EAT, as well as immunometabolic and secretory features of this tissue, could serve as a target for causal treatment of cardiac cachexia.

## Conflict of interest

None declared.

## Funding

Ministry of Health (AZV ČR 17‐28784A, AZV16‐27496A and NV19‐02‐00118) and the project for the development of research organization 00023001 IKEM—institutional support.

## Supporting information


**Data S1.** **Method S1** Untargeted metabolomic and lipidomic analysis of EAT
**Table S1.** PCR primers
**Table S2.** Expression of selected genes in EAT
**Table S3.** Drugs and other non‐physiological analytes in EAT extracts
**Table S4.** Cardiolipin and phosphatidylglycerol species levels in EAT
**Table S5.** Clinical variables, medication and plasma parameters in Cluster A4‐ and Cluster B4‐patients
**Table S6.** Expression of selected genes involved in cardiolipin synthesis in EAT
**Figure S1.** Chemical classes of all the analytes with known structure detected in EAT.
**Figure S2.** Correlations between levels of CLs and PGs in EAT.
**Figure S3.** Induction of EAT lipid metabolism linked to cachexia – formation and role of CL.

**Dataset S1.** All 750 analytes with known structure detected in EAT extracts from HF‐patients, including isobars.

**Dataset S2.** Analytes in EAT extracts with known structures and significantly different mean levels in (*A*) BW‐stable and cachectic, and (*B*) Cluster A4‐ and Cluster B4‐patients, respectively.
