# Supplementary material for: Dysregulation of epicardial adipose tissue in cachexia due to heart failure: the role of natriuretic peptides and cardiolipin
Source: J Cachexia Sarcopenia Muscle. 2020 Oct 20;11(6):1614–27. doi: 10.1002/jcsm.12631 (PMC7749591; doi:10.1002/jcsm.12631)
Supplement: Supplementary file 1 — Data S1. Method S1 Untargeted metabolomic and lipidomic analysis of EAT Table S1. PCR primers Table S2. Expression of selected genes in EAT Table S3. Drugs and other non‐physiological analytes in EAT extracts Table S4. Cardiolipin and phosphatidylglycerol species levels in EAT Table S5. Clinical variables, medication and plasma parameters in Cluster A4‐ and Cluster B4‐patients Table S6. Expression of selected genes involved in cardiolipin synthesis in EAT Figure S1. Chemical classes of all the analytes with known structure detected in EAT. Figure S2. Correlations between levels of CLs and PGs in EAT. Figure S3. Induction of EAT lipid metabolism linked to cachexia – formation and role of CL. [file JCSM-11-1614-s003.docx]

**Online supplementary material**

***Journal of Cachexia, Sarcopenia and Muscle***

**Dysregulation of epicardial adipose tissue in cachexia due to heart failure: the role of natriuretic peptides and cardiolipin**

Petra Janovska^2 *^, Vojtech Melenovsky^1 * †^**,** Michaela Svobodova^2^, Tereza Havlenova^1^, Helena Kratochvilova^1^, Martin Haluzik^1^, Eva Hoskova^1^, Terezie Pelikanova^1^, Josef Kautzner^1^, Luca Monzo^1^, Ivana Jurcova^1^, Katerina Adamcova^2^, Lucie Lenkova^2^, Jana Buresova^2^, Martin Rossmeisl^2^, Ondrej Kuda^2^, Tomas Cajka^2^, Jan Kopecky^2^ ^†^

**Contributed equally*

^1^Institute for Clinical and Experimental Medicine - IKEM, Prague, Czech Republic

^2^Institute of Physiology of the Czech Academy of Sciences, Prague, Czech Republic

†*Co-corresponding authors:*

Vojtech Melenovsky, MD, PhD
Department of Cardiology, IKEM

Vídeňská 1958/9, Prague 4, 140 28, Czech Republic

E-mail:[vojtech.melenovsky@ikem.cz](mailto:vojtech.melenovsky@ikem.cz)

Jan Kopecký, MD, PhD

Department of Adipose Tissue Biology

Institute of Physiology Czech Acad Sci

Vídeňská 1083, Prague 4, 140 20, Czech Republic

E-mail: jan.kopecky@fgu.cas.cz

**Method S1** Untargeted metabolomics and lipidomics analysis of EAT

EAT metabolomic and lipidomic profiling was conducted using a combined targeted and untargeted workflow for the lipidome, metabolome, and exposome analysis (LIMeX) [1, 2]. Extraction was carried out using a biphasic solvent system of cold methanol, methyl tert-butyl ether (MTBE), and water [3] with some modifications. In more detail, 10–25 mg of EAT samples were homogenized (1.5 min) with 275 µL methanol and 275 µL 10% methanol both containing internal standards using a grinder. Then, 1 mL of MTBE with internal standard was added, the tubes were shaken (1 min) and centrifuge (16,000 rpm, 5 min, 4°C). In total, 6 different LC-MS platforms were used for metabolomic and lipidomic profiling: (i) lipidomics of high-abundant triacylglycerols (TGs) in positive ion mode; (ii) lipidomics of low-abundant triacylglycerol estolides (TAG-EST) in positive ion mode; (iii) lipidomics of minor polar lipids in positive ion mode; (iv) lipidomics of minor polar lipids in negative ion mode; (v) metabolomics of polar metabolites and drugs in positive ion mode (BEH Amide platform); and (vi) metabolomics of polar metabolites in negative ion mode (HSS T3 platform).

For profiling of high-abundant triglycerides (TAG) in positive ion mode, 100 µL of upper organic phase was collected, resuspended using a chloroform/methanol/isopropanol (1:2:4) mixture, shaken (30 s), centrifuged (16,000 rpm, 5 min, 4°C) and the extract was further 100-fold diluted with methanol containing 12-[[(cyclohexylamino)carbonyl]amino]-dodecanoic acid (CUDA) internal standard. For low-abundant triacylglycerol estolides (TAG-EST) in positive ion mode, the resuspended extract in a chloroform/methanol/isopropanol mixture was diluted 10-fold with methanol and injected with modified mass range for data acquisition (see below). For profiling of minor, polar lipid species in positive and negative ion modes, 100 µL of upper organic phase was collected, resuspended using 80% methanol with CUDA internal standard, shaken (30 s), centrifuged (16,000 rpm, 5 min, 4°C) and used for LC-MS analysis. An aliquot of 70 µL of bottom aqueous phase was collected, evaporated, resuspended in 70 µL of an acetonitrile/water (80:20) mixture with CUDA and Val-Tyr-Val internal standards, shaken (30 s), centrifuged (16,000 rpm, 5 min, 4°C) and analyzed using HILIC metabolomics platform.

Another 70 µL aliquot of bottom aqueous phase was mixed with 210 µL of an isopropanol/acetonitrile (1:1) mixture, shaken (30 s), centrifuged (16,000 rpm, 5 min, 4°C), and the supernatant was evaporated, resuspended in 70 µL of 5% methanol/0.2% formic acid with CUDA and Val-Tyr-Val internal standards, shaken (30 s), centrifuged (16,000 rpm, 5 min, 4°C), and analyzed using HSS T3 metabolomics platform.

For LC-MS analysis, the systems consisted of a Vanquish UHPLC System (Thermo Fisher Scientific, Bremen, Germany) coupled to a QExactive Plus mass spectrometer (Thermo Fisher Scientific, Bremen, Germany) was used.

Lipids were separated on an Acquity UPLC BEH C18 column (50 × 2.1 mm; 1.7 μm) coupled to an Acquity UPLC BEH C18 VanGuard pre-column (5 × 2.1 mm; 1.7 μm) (Waters, Milford, MA, USA). The column was maintained at 65°C at a flow-rate of 0.6 mL/min. For LC–ESI(+)-MS analysis, the mobile phase consisted of (A) 60:40 (v/v) acetonitrile:water with ammonium formate (10 mM) and formic acid (0.1%) and (B) 90:10:0.1 (v/v/v) isopropanol:acetonitrile:water with ammonium formate (10 mM) and formic acid (0.1%). For LC–ESI(−)-MS analysis, the composition of the solvent mixtures were the same with the exception of the addition of ammonium acetate (10 mM) and acetic acid (0.1%) as mobile-phase modifier. Separation was conducted under the following gradient for LC–ESI(+)-MS: 0 min 15% (B); 0–1 min 30% (B); 1–1.3 min from 30% to 48% (B); 1.3–5.5 min from 48% to 82% (B); 5.5–5.8 min from 82% to 99% (B); 5.8–6 min 99% (B); 6–6.1 min from 99% to 15% (B); 6.1–7.5 min 15% (B). For LC–ESI(−)-MS, the following gradient was used: 0 min 15% (B); 0–1 min 30% (B); 1–1.3 min from 30% to 48% (B); 1.3–4.8 min from 48% to 76% (B); 4.8–4.9 min from 76% to 99% (B); 4.9–5.3 min 99% (B); 5.3–5.4 min from 99% to 15% (B); 5.4–6.8 min 15% (B). A sample volume of 0.5–5 μL was used for injection based on extract type. Sample temperature was maintained at 4°C.

The source and MS parameters were sheath gas pressure, 60 arbitrary units; aux gas flow, 25 arbitrary units; sweep gas flow, 2 arbitrary units; capillary temperature, 300°C; aux gas heater temperature, 370°C. For general lipidomics profiling the mass spectrometer was operated under following conditions: MS1 mass range, *m*/*z* 200–1700; MS1 resolving power, 35,000 FWHM (*m/z* 200); number of data-dependent scans per cycle, 3; MS/MS resolving power, 17,500 FWHM (*m/z* 200). For TAG-EST platform the MS1 mass range of *m*/*z* 1000–1600 was used. For ESI(+), a spray voltage of 3.6 kV and normalized collision energy of 20% was used while for ESI(−) a spray voltage of −3.0 kV and normalized collision energy of 10, 20 and 30% were set-up.

Polar metabolites were separated on an Acquity UPLC BEH Amide column (50 × 2.1 mm; 1.7 μm) coupled to an Acquity UPLC BEH Amide VanGuard pre-column (5 × 2.1 mm; 1.7 μm) (Waters, Milford, MA, USA). The column was maintained at 45°C at a flow-rate of 0.4 mL/min. The mobile phase consisted of (A) water with ammonium formate (10 mM) and formic acid (0.125%) and (B) acetonitrile:water (95/5) with ammonium formate (10 mM) and formic acid (0.125%). Separation was conducted under the following gradient: 0 min 100% (B); 0–1 min 100% (B); 1–3.9 min from 100% to 70% (B); 3.9–5.1 min from 70% to 30% (B); 5.1–6.4 min from 30% to 100%(B); 6.4–8.0 min 100% (B). A sample volume of 1 μL was used for injection. Sample temperature was maintained at 4°C. Polar metabolites were also separated on an Acquity UPLC HSS T3 column (50 × 2.1 mm; 1.7 μm) coupled to an Acquity UPLC HSS T3 VanGuard pre-column (5 × 2.1 mm; 1.7 μm) (Waters, Milford, MA, USA). The column was maintained at 45°C using a ramped flow-rate. The mobile phase consisted of (A) water with formic acid (0.2%) and (B) methanol with formic acid (0.1%). Separation was conducted under the following gradient: 0 min 1% (B) 0.3 mL/min; 0–0.5 min 1% (B) 0.3 mL/min; 0.5–2 min from 1% to 60% (B) 0.3 mL/min; 2–2.3 min from 60% to 95% (B) from 0.3 mL/min to 0.5 mL/min; 2.3–3.0 min 95% (B) 0.5 mL/min; 3.0–3.1 min from 95% to 1% (B) 0.5 mL/min; 3.1–4.5 min 1% (B) 0.5 mL/min; 4.5–4.6 min 1% (B) from 0.5 mL/min to 0.3 mL/min; 4.6–5.5 min 1% (B) 0.3 mL/min. A sample volume of 5 μL was used for injection. Sample temperature was maintained at 4°C.

The ESI source and MS parameters were: sheath gas pressure, 50 arbitrary units; aux gas flow, 13 arbitrary units; sweep gas flow, 3 arbitrary units; capillary temperature, 260°C; aux gas heater temperature, 425°C. For general metabolomics profiling the mass spectrometer was operated under following conditions: MS1 mass range, *m/z* 60–900; MS1 resolving power, 35,000 FWHM (*m/z* 200); number of data-dependent scans per cycle, 3; MS/MS resolving power, 17,500 FWHM (*m/z* 200). A spray voltage of 3.6 kV and −2.5 kV for ESI(+) and ESI(–), respectively, was used. For both platforms a normalized collision energy of 20, 30 and 40% were used.

LC-MS data from metabolomic and lipidomic profiling were processed through MS-DIAL v. 2.52 and 2.80 software [4]. Metabolites were annotated using in-house retention time–*m/z* library and using MS/MS libraries available from public sources (MassBank, MoNA, LipidBlast). Raw data were filtered using blank samples, serial dilution samples, and quality control (QC) pool samples with relative standard deviation (RSD) <30%, normalized using LOESS approach by means of QC pool samples injected regularly between 10 actual samples, and further normalized using the amount of EAT samples taken for analysis. Samples were randomized across the platform run.

**References**

1. Paluchova V, Oseeva M, Brezinova M, Cajka T, Bardova K, Adamcova K, et al. Lipokine 5-PAHSA Is Regulated by Adipose Triglyceride Lipase and Primes Adipocytes for De Novo Lipogenesis in Mice. Diabetes 2020; 69: 300-12.

2. Brezinova M, Cajka T, Oseeva M, Stepan M, Dadova K, Rossmeislova L, et al. Exercise training induces insulin-sensitizing PAHSAs in adipose tissue of elderly women. Biochim Biophys Acta Mol Cell Biol Lipids 2020; 1865: 158576.

3. Cajka T, Smilowitz JT, and Fiehn O. Validating Quantitative Untargeted Lipidomics Across Nine Liquid Chromatography-High-Resolution Mass Spectrometry Platforms. Anal Chem 2017; 89: 12360-8.

4. Tsugawa H, Cajka T, Kind T, Ma Y, Higgins B, Ikeda K, et al. MS-DIAL: data-independent MS/MS deconvolution for comprehensive metabolome analysis. Nat Methods 2015; 12: 523-6.

T**able S1 PCR primers**

| **Gene Abbreviation** | **Gene Name** | **ID** | **5'primer** | **3'primer** |
| --- | --- | --- | --- | --- |
| ACADL | Long-chain acyl-coenzyme A dehydrogenase | 33 | CCTGCCCATGGTATTAGCCTTTTTCT | TAGTTCTGCGGTATCCTGGGCTTTTA |
| ACSL1 | Acyl-CoA synthetase long chain family member 1 | 2180 | CATGCGAAGTGAGCCTGTTG | GCACAGTTCCTCAAACGACC |
| ADIPOQ | Adiponectin | 9370 | TCACAACAGAAAACCCAAAAT | GCGGAAGGCAGAACAGAAG |
| ADORA1 | Adenosine A1 receptor | 11539 | ACATCCAGTGGGGTCTCAGT | CTCCTAGTGGAGGGACCACA |
| ATGL | Adipose triglyceride lipase, also called PNPLA2 | 57104 | TCGCAGCTGCCCGGAGAAGAT | AGCAAGCGGATGGTGAAGGACAGAG |
| CIDEA | Cell death-inducing DNA fragmentation factor, alpha subunit-like effector A | 1149 | CCCAAGGACTTCATCGGCT | GCACCCGGAGCATGTATGTG |
| CRLS1 | Cardiolipin synthase 1 | 54675 | GGGGGCATGTGGCTGGCTATT | TGTGCCTCTTGGGTGGCTCAG |
| DGAT1 | Diacylglycerol O-acyltransferase 1 | 8694 | AAGTGCTGTCCAGTGACCTC | TCCACACAGCTCTGGCACTC |
| DGAT2 | Diacylglycerol O-acyltransferase 2 | 84649 | GGCCACCTGCTTTGGAACTA | TGGATGCAGCACAGACTCAG |
| FABP1 | Fatty acid binding protein 1 | 2168 | TGCGTCATGAAAGGCGTCACT | GTCCAGGTCAACGTCCCTTGG |
| FAS | Fatty acid synthase | 2194 | AGCGCCCGCTCTGGTTCATCTG | GGGGGCAGCGCTGTTTACACTCC |
| GAPDH | Glyceraldehyde 3-phosphate dehydrogenase | 2597 | ACAGTCAGCCGCATCTTCTTTTG | GACGTACTCAGCGCCAGCATC |
| HPRT | Hypoxanthine-guanine phosphoribosyltransferase | 3251 | AGCCCTGGCGTCGTGATTAG | TGATGGCCTCCCATCTCCTT |
| HSL | Hormone-sensitive lipase, also called LIPE | 3991 | GCTGCGTGGGGCTGAGTTTGAG | GTGTGGGCCAGTGGGGGTGAGAT |
| LCLAT1 | Lysocardiolipin acyltransferase 1 | 253558 | TAGCCCTGACTATGATGCCCCTGT | TAACACCAACAGGCTCCCTCCG |
| LEP | Leptin | 3952 | GGGAACCCTGCTTGCACTTTGTA | CCTGTTGGCTGTTATGGTCTTATGTATTTT |
| NPRA | Natriuretic peptide receptor 1 | 4881 | AGAGCAAGGACACACCGTTT | CCACTCCTCAGGGCAATCTC |
| NPRC | Natriuretic peptide receptor 3, also called clearance receptor | 4883 | CAGGTCAAAATGCGTAGATGCT | CTGTGGTCAGTCTTGAGGGAG |
| PDK4 | Pyruvate dehydrogenase kinase 4 | 5166 | CGGCTTGCCAATTTCTCGTCT | GCCACTTCTTTTGCCAGGTTCTTTG |
| PEPCK | Phosphoenolpyruvate carboxykinase | 5105 | CTGGGAGAAGGAGGTGGAAGACA | TTATGGATGGGAAAGGGAATGAT |
| PGC-1α | Peroxisome proliferative-activated receptor y coactivator 1α, also PPARGC1A | 10891 | TGGGGTCAGAGGAAGAGATAAAGT | TCATGGAGCAATAAAGCGAAGAGTA |
| PGS1 | Phosphatidylglycerophosphate synthase 1 | 9489 | CCACCTTCGAGCAGCCGAGT | CAAGGCCATTCCTGTCTGTCCTC |
| PLIN1 | Perilipin 1 | 5346 | CCTGAAGGGCGTTACTGACA | CTGGTGGGTTGTCGATGTCC |
| PPIA | Peptidylprolyl isomerase A | 5478 | GTATAAAAGGGGCGGGAGGC | CTGCAAACAGCTCAAAGGAGAC |
| PPARα | Peroxisome proliferator activated receptor α | 5465 | CAAAGCCCGGGTCATCCTCTCA | CCAGCATCCCGTCTTTGTTCATCA |
| PPARγ | Peroxisome proliferator activated receptor γ | 5468 | GCCTTGCAGTGGGGATGTCTCA | GATGCGGATGGCCACCTCTTTG |
| PTGDS | Prostaglandin D2 synthase | 5730 | TTCCTGCCCCAAACCGATAAGTG | TCGGGGAAGGAACAGAGCAGAGA |
| TNFα | Tumor necrosis factor α | 7124 | CGCCACCACGCTCTTCTGC | GCTTGAGGGTTTGCTACA |
| ZAG | Zinc-binding alpha-2-glycoprotein 1 | 563 | CCTGCGACTCTGCGGAAATA | TGGGTAGAAGTCGTAGGCCA |

Expression of selected genes in EAT was evaluated using total RNA isolated from the tissue (RNeasy Lipid Tissue Mini Kit; Qiagen, Valencia, CA, USA), and primers shown in the table. ID, gene identifier from NCBI.

**Table S2 Expression of selected genes in EAT**

| **Gene name** | **BW-stable** | | | **Cachexia** | | | **FC** | ***P*-value** | **Cluster A4** | | | **Cluster B4** | | | **FC** | ***P*-value** |
| --- | --- | --- | --- | --- | --- | --- | --- | --- | --- | --- | --- | --- | --- | --- | --- | --- |
|  |  |  |  |  |  |  |  |  |  |  |  |  |  |  |  |  |
|  | *n* = 35 | | | *n* = 17 | | | Cachexia/ BW-stable |  | *n* = 39 | | | *n* = 13 | | | B4/A4 |  |
| NPRA | 1.59 | ± | 0.10 | 1.89 | ± | 0.33 | 1.19 | 0.404 | 1.57 | ± | 0.19 | 1.73 | ± | 0.16 | 1.10 | 0.534 |
| NPRC | 2.07 | ± | 0.35 | 0.88 | ± | 0.16 | 0.42 | **0.003** | 3.49 | ± | 0.71 | 1.08 | ± | 0.15 | 0.31 | **0.006** |
| ADORA1 | 1.37 | ± | 0.11 | 2.35 | ± | 0.53 | 1.71 | 0.092 | 1.19 | ± | 0.20 | 1.86 | ± | 0.25 | 1.56 | **0.041** |
| PTGDS | 1.41 | ± | 0.12 | 1.65 | ± | 0.21 | 1.17 | 0.285 | 1.52 | ± | 0.21 | 1.48 | ± | 0.12 | 0.98 | 0.883 |
| ATGL | 1.74 | ± | 0.13 | 1.91 | ± | 0.34 | 1.10 | 0.654 | 1.68 | ± | 0.23 | 1.83 | ± | 0.17 | 1.09 | 0.642 |
| HSL | 1.37 | ± | 0.14 | 2.03 | ± | 0.45 | 1.48 | 0.183 | 1.51 | ± | 0.31 | 1.61 | ± | 0.22 | 1.06 | 0.827 |
| PLIN1 | 1.62 | ± | 0.17 | 1.95 | ± | 0.40 | 1.20 | 0.456 | 1.82 | ± | 0.33 | 1.70 | ± | 0.20 | 0.94 | 0.773 |
| CIDEA | 2.22 | ± | 0.20 | 2.36 | ± | 0.43 | 1.06 | 0.780 | 2.10 | ± | 0.25 | 2.32 | ± | 0.25 | 1.10 | 0.539 |
| ZAG | 1.48 | ± | 0.17 | 2.35 | ± | 0.45 | 1.10 | 0.654 | 1.68 | ± | 0.23 | 1.83 | ± | 0.17 | 1.09 | 0.642 |
|  |  |  |  |  |  |  |  |  |  |  |  |  |  |  |  |  |
| FAS | 0.74 | ± | 0.08 | 2.13 | ± | 0.58 | 2.89 | **0.030** | 0.62 | ± | 0.12 | 1.39 | ± | 0.28 | 2.25 | **0.014** |
| PEPCK | 2.16 | ± | 0.21 | 3.36 | ± | 0.66 | 1.60 | 0.087 | 1.96 | ± | 0.37 | 2.77 | ± | 0.33 | 1.37 | 0.149 |
| DGAT1 | 1.65 | ± | 0.16 | 2.69 | ± | 0.42 | 1.63 | **0.030** | 1.60 | ± | 0.21 | 2.11 | ± | 0.23 | 1.32 | 0.110 |
| DGAT2 | 1.46 | ± | 0.17 | 1.86 | ± | 0.35 | 1.28 | 0.302 | 1.26 | ± | 0.22 | 1.70 | ± | 0.20 | 1.35 | 0.147 |
| FABP1 | 1.61 | ± | 0.17 | 2.04 | ± | 0.35 | 1.27 | 0.280 | 1.52 | ± | 0.25 | 1.83 | ± | 0.20 | 1.21 | 0.405 |
|  |  |  |  |  |  |  |  |  |  |  |  |  |  |  |  |  |
| PPARα | 1.41 | ± | 0.12 | 1.69 | ± | 0.19 | 1.20 | 0.210 | 1.45 | ± | 0.20 | 1.52 | ± | 0.12 | 1.04 | 0.797 |
| PPARγ | 1.24 | ± | 0.14 | 1.45 | ± | 0.25 | 1.17 | 0.438 | 1.70 | ± | 0.30 | 1.18 | ± | 0.13 | 0.70 | 0.075 |
| PGC-1α | 1.01 | ± | 0.11 | 1.31 | ± | 0.25 | 1.30 | 0.286 | 1.17 | ± | 0.22 | 1.09 | ± | 0.13 | 0.93 | 0.749 |
| PDK4 | 0.92 | ± | 0.10 | 1.12 | ± | 0.19 | 1.21 | 0.333 | 1.03 | ± | 0.19 | 0.98 | ± | 0.11 | 0.95 | 0.817 |
| ACSL1 | 1.16 | ± | 0.11 | 1.67 | ± | 0.31 | 1.44 | 0.143 | 1.13 | ± | 0.21 | 1.39 | ± | 0.16 | 1.24 | 0.383 |
| ACADL | 1.63 | ± | 0.14 | 1.77 | ± | 0.33 | 1.08 | 0.712 | 1.77 | ± | 0.24 | 1.65 | ± | 0.17 | 0.93 | 0.708 |
|  |  |  |  |  |  |  |  |  |  |  |  |  |  |  |  |  |
| ADIPOQ | 1.16 | ± | 0.22 | 1.33 | ± | 0.35 | 1.15 | 0.655 | 1.36 | ± | 0.32 | 1.16 | ± | 0.22 | 0.86 | 0.647 |
| LEP | 1.49 | ± | 0.15 | 1.19 | ± | 0.25 | 0.80 | 0.297 | 1.97 | ± | 0.24 | 1.20 | ± | 0.14 | 0.61 | **0.009** |
| TNFα | 2.04 | ± | 0.40 | 1.70 | ± | 0.58 | 0.86 | 0.689 | 1.89 | ± | 0.79 | 1.94 | ± | 0.35 | 1.03 | 0.949 |

Levels (A.U.) of the transcript are shown for two subroups of the patients split based on (i) BW trajectories during the previous 6 months (BW-stable vs. Cachexia; see *Table* 1 normalized to geometric mean of 3 housekeeping genes, as shown also in *Figure* 1, but without the cube root transformation of the data; and (ii) hierarchial clustering of their EAT metabolome (Cluster A4 vs. Cluster B4; see *Figure* 5D). Data are means ± SE; Student’s *t*-test and considered as significant when *P* ≤ 0.05. FC, fold-change difference of the means.

**Table S3 Drugs and other non-physiological analytes in EAT extracts**

| **Metabolite name** | **Chemical class** |
| --- | --- |
| 4-Acetamidoantipyrine | Drug |
| 4-Aminoantipyrine | Drug |
| 4-Aminomethylcyclohexanecarboxylic acid (tranexamic acid) | Drug |
| 4-Formylaminoantipyrine | Drug |
| Acetaminophen glucuronide | Drug |
| Acetyl-Sulfamethoxazole (metabolite of sulfamethoxazole) | Drug |
| alpha-Hydroxymetoprolol | Drug |
| Amiodarone | Drug |
| Azithromycin | Drug |
| Cefuroxime | Drug |
| Cotinine (metabolite of nicotine) | Drug |
| Descladinoseazithromycin | Drug |
| Desethylamiodarone | Drug |
| Desmethylcitalopram | Drug |
| Diazepam | Drug |
| Gabapentin | Drug |
| Iomeprol | Drug |
| Lidocaine | Drug |
| Metformin | Drug |
| Metoclopramide | Drug |
| Metoprolol | Drug |
| Metoprolol acid | Drug |
| Midazolam | Drug |
| N,N-Diethyl-2-aminoethanol | Drug |
| Omeprazole | Drug |
| Omeprazole sulfone (metabolite of omeprazole) | Drug |
| Pantoprazole | Drug |
| Sertraline | Drug |
| Trazodone | Drug |
| Warfarin | Drug |
| Zolpidem | Drug |
| Ergothioneine | Other analyte |
| Gluconic acid | Other analyte |
| Hippurate | Other analyte |
| Histamine | Other analyte |
| Lenticin | Other analyte |
| N,N-Dimethyldodecylamine N-oxide | Other analyte |
| PEtOH 34:1 (16:0-18:1) | Other analyte |
| PEtOH 36:2 (18:1-18:1) | Other analyte |
| PEtOH 36:4 (16:0-20:4) | Other analyte |
| PEtOH 38:4 (18:0-20:4) | Other analyte |
| Piperine | Other analyte |
| Sebacic acid | Other analyte |
| Stachydrine (proline betaine) | Other analyte |
| Thiamine | Other analyte |
| Threonic acid | Other analyte |
| Trigonelline | Other analyte |

List of non-physiological analytes, namely drugs (*n* = 31) and other non-physiological analytes (other analytes, *n* = 16) most probably coming from nutrition, which were detected among all the other EAT analytes (see *Figure* S1 and *Dataset* 1).

**Table S4 Cardiolipin and phosphatidylglycerol species levels in EAT**

|  | **All patients** | | | **BW-stable** | | | **Cachexia** | | | **FC** | ***P*-value** | **Cluster A4** | | | **Cluster B4** | | | **FC** | ***P*-value** |
| --- | --- | --- | --- | --- | --- | --- | --- | --- | --- | --- | --- | --- | --- | --- | --- | --- | --- | --- | --- |
|  | *n* = 51 | | | *n* = 34 | | | *n* = 17 | | | Cachexia/ BW-stable |  | *n* = 39 | | | *n* = 13 | | | B4/A4 |  |
| **Cardiolipins** | |  |  |  |  |  |  |  |  |  |  |  |  |  |  |  |  |  |  |
| ^a^CL 70:6 | 1589 | ± | 244 | 1294 | ± | 275 | 2074 | ± | 478 | 1,53 | 0.168 | 45 | ± | 17 | 2104 | ± | 276 | 47.25 | **<0.001** |
| CL 70:7 | 4119 | ± | 532 | 3538 | ± | 533 | 4218 | ± | 584 | 1.04 | 0.877 | 1208 | ± | 133 | 5090 | ± | 629 | 4.21 | **<0.001** |
| CL 72:7 | 6250 | ± | 1100 | 4982 | ± | 595 | 5756 | ± | 592 | 0.89 | 0.671 | 3185 | ± | 355 | 7272 | ± | 1410 | 2.28 | **0.008** |
| CL 72:8 | 22191 | ± | 6866 | 14328 | ± | 1891 | 18105 | ± | 4364 | 0.75 | 0.581 | 10244 | ± | 1090 | 26173 | ± | 8968 | 2.55 | 0.090 |
| **Phosphatidylglycerols** | | | |  |  |  |  |  |  |  |  |  |  |  |  |  |  |  |  |
| PG 34:1 (1) | 3282 | ± | 751 | 2266 | ± | 330 | 5343 | ± | 2159 | 2.34 | 0.179 | 2274 | ± | 663 | 3618 | ± | 961 | 1.59 | 0.266 |
| PG 34:1 (2) | 14987 | ± | 2571 | 12107 | ± | 986 | 13558 | ± | 2090 | 0.86 | 0.620 | 9504 | ± | 811 | 16814 | ± | 3333 | 1.77 | **0.042** |
| PG 34:2 (1) | 3264 | ± | 683 | 2389 | ± | 414 | 5097 | ± | 1878 | 2.15 | 0.174 | 1420 | ± | 390 | 3879 | ± | 870 | 2.73 | **0.015** |
| PG 34:2 (2) | 1720 | ± | 241 | 1423 | ± | 139 | 1675 | ± | 202 | 0.96 | 0.867 | 1148 | ± | 116 | 1911 | ± | 310 | 1.67 | **0.028** |
| PG 36:1 | 3096 | ± | 295 | 2730 | ± | 191 | 3106 | ± | 356 | 1.00 | 0.979 | 2280 | ± | 283 | 3368 | ± | 367 | 1.48 | **0.027** |
| PG 36:2 | 19369 | ± | 3578 | 14850 | ± | 1938 | 28667 | ± | 10066 | 1.93 | 0.195 | 12450 | ± | 3510 | 21675 | ± | 4518 | 1.74 | 0.122 |
| PG 36:3 | 10904 | ± | 1814 | 8394 | ± | 1104 | 15779 | ± | 4975 | 1.85 | 0.172 | 7008 | ± | 2142 | 12203 | ± | 2248 | 1.74 | 0.111 |
| PG 36:4 (1) | 2146 | ± | 362 | 1620 | ± | 232 | 3128 | ± | 977 | 1.87 | 0.164 | 1179 | ± | 398 | 2468 | ± | 447 | 2.09 | **0.042** |
| PG 36:4 (2) | 863 | ± | 148 | 661 | ± | 78 | 922 | ± | 240 | 1.10 | 0.786 | 342 | ± | 44 | 1036 | ± | 187 | 3.03 | **<0.001** |
| PG 36:4 (3) | 1044 | ± | 102 | 946 | ± | 105 | 1065 | ± | 151 | 1.03 | 0.884 | 787 | ± | 178 | 1129 | ± | 118 | 1.43 | 0.135 |
| PG 38:4 | 806 | ± | 160 | 621 | ± | 113 | 1162 | ± | 430 | 1.84 | 0.249 | 539 | ± | 217 | 895 | ± | 197 | 1.66 | 0.247 |
| PG 38:5 | 2131 | ± | 362 | 1655 | ± | 230 | 2979 | ± | 987 | 1.73 | 0230 | 1320 | ± | 428 | 2401 | ± | 448 | 1.82 | 0.097 |
| PG 40:6 | 899 | ± | 173 | 724 | ± | 142 | 1248 | ± | 444 | 1.71 | 0.278 | 466 | ± | 229 | 1043 | ± | 210 | 2.24 | 0.080 |
| PG 40:7 | 3673 | ± | 769 | 2615 | ± | 404 | 5265 | ± | 2121 | 1.82 | 0.291 | 2166 | ± | 597 | 4175 | ± | 982 | 1.93 | 0.093 |
| PG 40:8 | 1321 | ± | 272 | 926 | ± | 155 | 1745 | ± | 670 | 1.56 | 0.387 | 672 | ± | 215 | 1537 | ± | 345 | 2.29 | **0.042** |

Levels (A.U.) of the analytes in EAT. For the source data, see Supplementary material online, *Dataset* S2. Data are shown for all the patients, or two subroups of the patients split based on (i) BW trajectories during the previous 6 months (BW-stable vs. Cachexia; see *Table* 1; and (ii) hierarchial clustering of their EAT metabolome (Cluster A4 vs. Cluster B4; see *Figure* 5D). Data are means ± SE; Student’s *t*-test and considered as significant when *P* ≤ 0.05. FC, fold-change difference of the means.

^a^ Sum of two isobars detected, (1) [16:1-18:2-18:1-18:2], representing the more abundant form, and (2) [16:1-18:1-18:2-18:2]. CL 70:7, [16:1-18:2-18:2-18:2]; CL 72:7, [18:1-18:2-18:2-18:2]; CL 72:8, [18:2-18:2-18:2-18:2]; PG 34:1 (1), [16:0-18:1]; PG 34:1 (2), [16:0-18:1]; PG 34:2 (1), [16:1-18:1]; PG 34:2 (2), [16:0-18:2]; PG 36:1, [18:0-18:1]; PG 36:2, [18:1-18:1]; PG 36:3, [18:1-18:2]; PG 36:4 (1), [18:2-18:2]; PG 36:4 (2), [18:2-18:2]; PG 36:4 (3), [16:0-20:4]; PG 38:4, [18:1-20:3]; PG 38:5, [18:1-20:4]; PG 40:6, [18:1-22:5]; PG 40:7, [18:1-22:6]; PG 40:8, [18:2-22:6].

**Table S5 Clinical variables, medication and plasma parameters in Cluster A4- and Cluster B4-patients**

|  | **Cluster A4** | | | **Cluster B4** | | | ***P*-value** |
| --- | --- | --- | --- | --- | --- | --- | --- |
|  | *n* = 13 | | | *n* = 39 | | |  |
| Age | 57 | ± | 9 | 53 | ± | 12 | 0.428 |
| Gender (M; %) | 92 | | | 56 | | | 0.290 |
| BMI | 28 | ± | 4 | 24 | ± | 4 | **0.006** |
| BW change (%) | -0.1 | ± | 3.3 | -6.4 | ± | 6.1 | **<0.001** |
| NYHA class | 3.1 | ± | 0.2 | 3.5 | ± | 0.6 | **0.001** |
| Non-ischemic HF (%) | 62 | | | 62 | | | 0.736 |
| HF duration (years) | 6.7 | ± | 3.6 | 4.7 | ± | 4.1 | 0.135 |
| Inotrope therapy (prior Tx; %) | 0 | | | 28 | | | **0.031** |
| **Echocardiography** |  |  |  |  |  |  |  |
| LV EF (%) | 22 | ± | 4 | 20 | ± | 7 | 0.239 |
| LVED diameter (mm) | 75 | ± | 10 | 73 | ± | 10 | 0.534 |
| MiR grade (0-4) | 2.2 | ± | 1.2 | 2.5 | ± | 1.1 | 0.468 |
| TriR (grade 0-4) | 2.0 | ± | 0.9 | 2.1 | ± | 1.0 | 0.801 |
| RVD1 diameter (mm) | 45 | ± | 9 | 46 | ± | 8 | 0.507 |
| RV dysfunction grade (0-4) | 1.2 | ± | 0.8 | 1.7 | ± | 1.0 | 0.075 |
| TAPSE (mm) | 17 | ± | 4 | 13 | ± | 4 | **0.014** |
| Epicardial fat thickness (mm) | 3.4 | ± | 1.6 | 2.4 | ± | 1.1 | **0.034** |
| **Hemodynamics** |  |  |  |  |  |  |  |
| Heart rate (s) | 74 | ± | 12 | 78 | ± | 14 | 0.749 |
| Mean blood pressure (mm Hg) | 88 | ± | 9 | 84 | ± | 10 | 0.216 |
| Cardiac output (L/min) | 3.7 | ± | 0.7 | 3.5 | ± | 0.8 | 0.259 |
| PA wedge pressure (mm Hg) | 25 | ± | 8 | 23 | ± | 8 | 0.619 |
| **Medication** |  |  |  |  |  |  |  |
| Furosemide (mg/day) | 143 | ± | 107 | 176 | ± | 179 | 0.427 |
| β-blockers (mg/day) | 88 | ± | 49 | 24 | ± | 40 | **<0.001** |
| ACE/ARB-inhibitors (mg/day) | 2.9 | ± | 2.8 | 0.9 | ± | 1.3 | **0.026** |
| **Plasma parameters** |  |  |  |  |  |  |  |
| BNP (pg/mL) | 897 | ± | 1044 | 1842 | ± | 1272 | **0.019** |
| Creatinine (µmol/L) | 108 | ± | 30 | 109 | ± | 46 | 0.914 |
| Total protein (g/L) | 62 | ± | 7 | 62 | ± | 6 | 0.757 |
| C reactive protein (g/L) | 7.4 | ± | 9.0 | 9.5 | ± | 13.0 | 0.598 |
| Hemoglobin (g/L) | 131 | ± | 10 | 124 | ± | 16 | 0.075 |
| Total cholesterol (mmol/L) | 4.0 | ± | 1.2 | 3.6 | ± | 1.0 | 0.354 |
| Triglycerides (mmol/L) | 1.6 | ± | 0.8 | 1.2 | ± | 0.7 | 0.139 |
| TSH (mUI/L) | 3.7 | ± | 4.5 | 4.0 | ± | 3.0 | 0.897 |
| fT3 (pmol/L ) | 3.5 | ± | 0.5 | 3.4 | ± | 0.9 | 0.405 |
| fT4 (pmol/L ) | 14 | ± | 3 | 15 | ± | 4 | 0.486 |
| Cortizol (nmol/L) | 450 | ± | 194 | 431 | ± | 257 | 0.809 |
| Fasting plasma glucose (mmol/L) | 5.8 | ± | 0.9 | 5.8 | ± | 1.3 | 0.943 |
| Fasting plasma insulin (µIU/mL) | 5.5 | ± | 5.3 | 3.7 | ± | 3.1 | 0.382 |
| HOMA-IR | 1.4 | ± | 1.5 | 0.9 | ± | 0.8 | 0.357 |
| Hemoglobin A1C (mmol/mol) | 48 | ± | 13 | 49 | ± | 10 | 0.821 |
| Bilirubin (µmol/L) | 18 | ± | 8 | 23 | ± | 12 | 0.083 |
| Sodium (mmol/L) | 138 | ± | 3 | 134 | ± | 4 | **0.005** |
| Potassium (mmol/L) | 4.0 | ± | 0.6 | 4.2 | ± | 0.6 | 0.687 |

Data are shown for two subroups of the patients split based hierarchial clustering of their EAT metabolome (Cluster A4 vs. Cluster B4; see *Figure* 5D). Data are means ± SD, Student’s *t*-test and considered as significant when *P* ≤ 0.05. For abbreviations and related data, see *Tables* 1 and 2.

**Table S6 Expression of selected genes involved in cardiolipin synthesis in EAT**

| **Gene name** | **BW-stable** | | | **Cachexia** | | | **FC** | ***P*-value** | **Cluster A4** | | | **Cluster B4** | | | **FC** | ***P*-value** |
| --- | --- | --- | --- | --- | --- | --- | --- | --- | --- | --- | --- | --- | --- | --- | --- | --- |
|  | *n* = 35 | | | *n* = 17 | | | Cachexia/ BW-stable |  | *n* = 39 | | | *n* = 13 | | | B4/A4 |  |
| PGS1 | 0.14 | ± | 0.05 | 0.12 | ± | 0.02 | 0.96 | 0.753 | 0.77 | ± | 0.08 | 0.93 | ± | 0.07 | 1.21 | 0.132 |
| CRLS1 | 0.83 | ± | 0.13 | 1.01 | ± | 0.17 | 1.23 | 0.405 | 0.59 | ± | 0.06 | 0.99 | ± | 0.14 | 1.66 | 0.011 |
| LCLAT1 | 2.54 | ± | 0.25 | 2.53 | ± | 0.61 | 0.99 | 0.984 | 2.11 | ± | 0.26 | 2.68 | ± | 0.33 | 1.27 | 0.182 |

Levels (A.U.) of the transcrip are shown for two subroups of the patients split based on (i) BW trajectories during the previous 6 months (BW-stable vs. Cachexia; see *Table* 1; and (ii) hierarchial clustering of their EAT metabolome (Cluster A4 vs. Cluster B4; see *Figure* 5D). Data are means ± SE; Student’s *t*-test and considered as significant when *P* ≤ 0.05. FC, fold-change difference of the means.

**Figure S1** Chemical classes of all the analytes with known structure detected in EAT. All 750 known analytes detected. Chemical classification was performed using the MetaboAnalyst v4.0 platform. Yellow, lipids; blue, polar compounds; grey, drugs and other non-physiological analytes (*n* = 48; see Supplementary material online, *Table* S3). Other lipids: bis(monoacylglycero)phosphate, cardiolipin, cholesterol, diacylglycerol, monoacylglycerol, monosialodihexosylganglioside. ACar, acylcarnitine; Cer, ceramide; FA, fatty acid; GM3, monosialodihexosylganglioside; PC, phosphatidylcholine; PE, phosphatidylethanolamine; PG, phosphatidylglycerol; PI, phosphatidylinositol; PS, phosphatidylserine; SM, sphingomyelin; TAG, triacylglycerol.


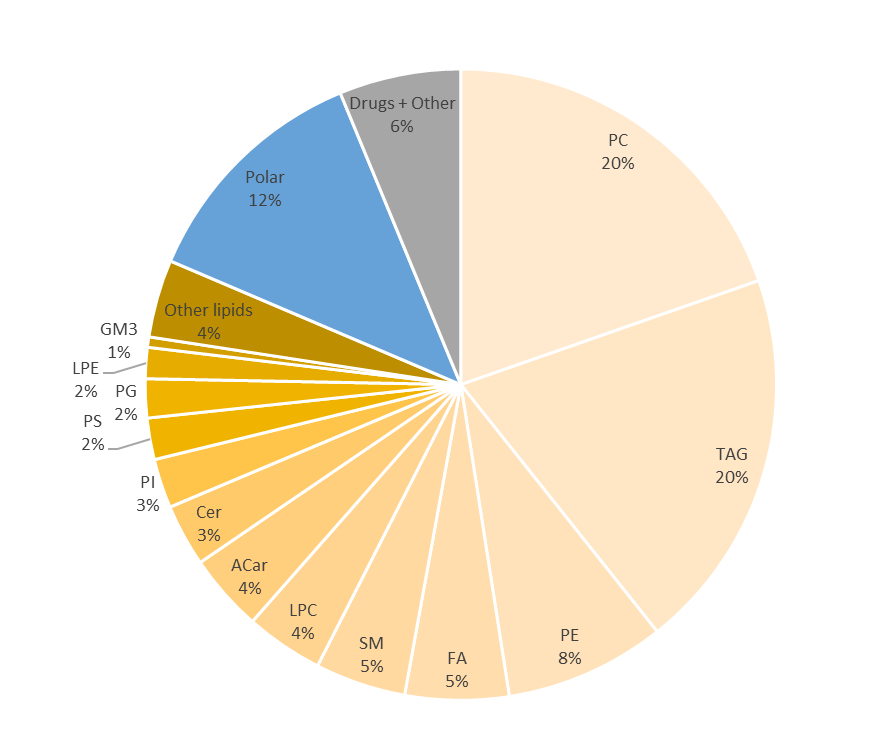


**Figure S2** Correlations between levels of CLs and PGs in EAT. For the source data, see Supplementary material online, *Dataset* 1. See also *Figure* 5. To assess the correlations between various analytes, Spearman's rank correlation coefficient (*r*) were calculated and indicated in the heatplot.

**Figure S3** Induction of EAT lipid metabolism linked to cachexia – formation and role of CL.

*Figure* 3 was extended to suggest the mechanisms behind enhaced formation of CL during pathologically overactivated lipolysis and its link with uncoupling of oxidative phosphorylation (OXPHOS) in adipocytes in cachexia. See also *Figure* 7. Higher rate of formation of phopshatidylglycerol (PG) species from glycerol-3-phosphtate (Glycerol-3P), the building blocks in CL synthesis, as well as dysregulation of genes engaged in synthesis of CL, i.e., PG synthase 1 (PGS1) and CL synthase 1 (CRLS1), and in the remodeling of composition of the acyl side chains of CL, i.e., acyl-CoA:lysocardiolipin acyltransferase 1 (LCLAT1) are probably involved. Levels of PGs might be elevated reflecting the sequenc e of events: (i) excessive activation of lipolysis, (ii) partial uncoupling of OXPHOS in response to the elevated intracellular fatty acids (FA) levels [1] – these levels increase in spite of quantitatively less important decrease in *de novo* FA synthesis (DNL) due to the lower rate of formation extramitochondrial acetyl-CoA by ‘pyruvate cycle’ (broken black lines; see ref. [2]), (iii) inhibiton of the ATP-demanding formation of triacylgycerol (TAG) starting from synthesis of monoacylgycerols (MAG); (vi) redirection of Glycerol-3P flux from MAG synthesis to formation of PG; (vii) OXPHOS uncoupling induced by CL. For other abbrevitaions, see *Figure* 3.

**
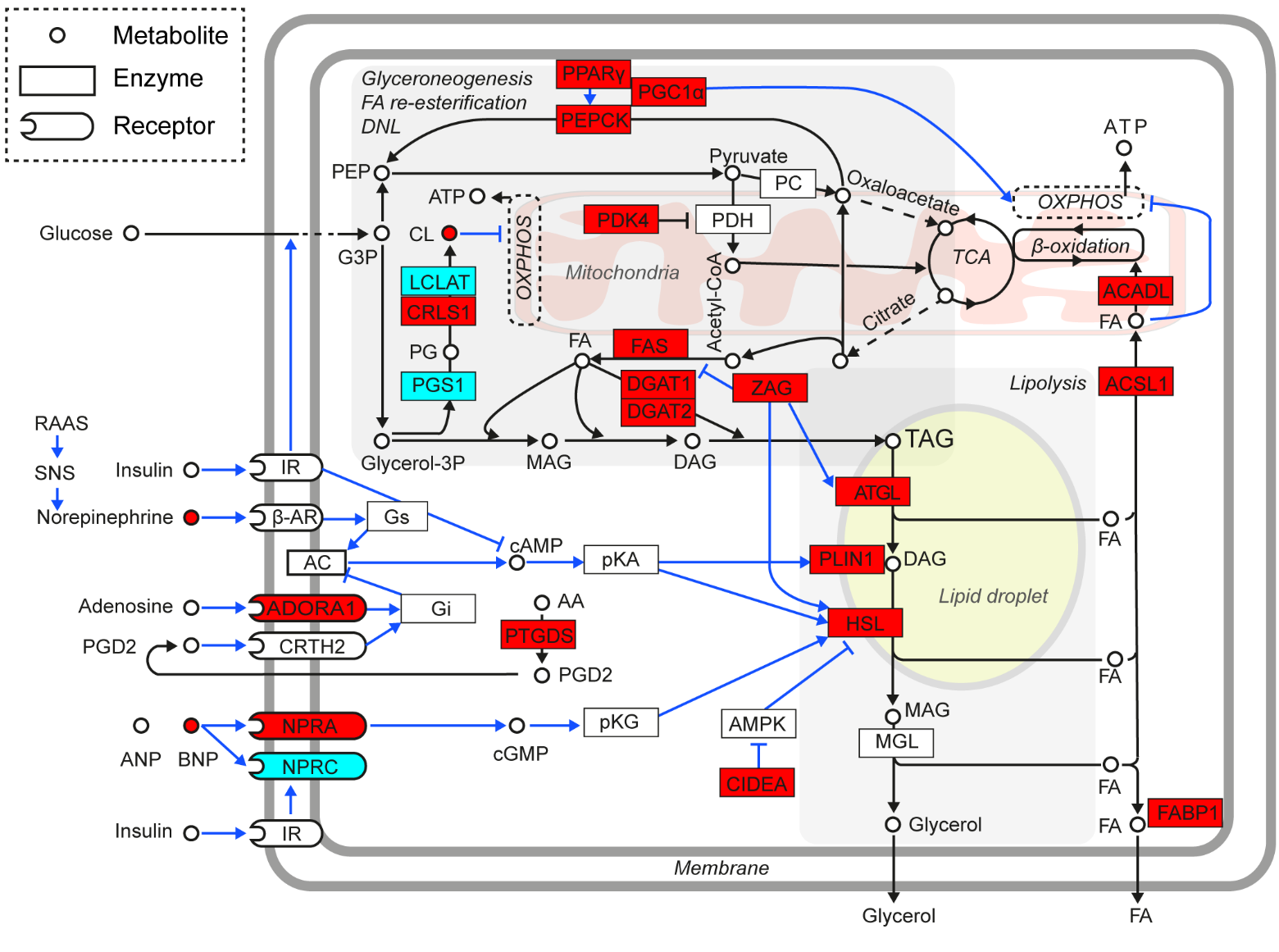
**

**References**

1. Yehuda-Shnaidman E, Buehrer B, Pi J, Kumar N, and Collins S. Acute stimulation of white adipocyte respiration by PKA-induced lipolysis. Diabetes 2010; **59**: 2474-83.

2. Rossmeisl M, Syrovy I, Baumruk F, Flachs P, Janovska P, and J. K. Decreased fatty acid synthesis due to mitochondrial uncoupling in adipose tissue. FASEB J 2000; **14**: 1793-800.

**Dataset S1** All 750 analytes with known structure detected in EAT extracts from HF-patients, including isobars; see also *Figure* S1.

**Dataset S2** Analytes in EAT extracts with known structures and significantly different mean levels in (*A*) BW-stable and cachectic, and (*B*) Cluster A4- and Cluster B4-patients, respectively. Comparisons were analyzed using Student’s *t*-test and considered as significant when *P* ≤ 0.05. The Benjamin-Hochberg false discovery rate (FDR) procedure was used on analyte levels to control for multiple testing, with a significance level of q <0.05. (*A*) Analytes in EAT extracts in BW-stable (*n* = 35) and cachectic (*n* = 17) patients. In total, 44 analytes discriminated between the two groups; however, only if FDR procedure was not applied. (*B*) Samples from HF-patients grouped in Cluster A4 (*n* = 13) and Cluster B4 (*n* = 39) that appeared in hierarchial cluster of EAT metabolome (*Figure* 5D) were compared. In total, 186 analytes with significantly different levels between the two groups were identified; FDR procedure was used.
